# GreedyMini: generating low-density DNA minimizers

**DOI:** 10.1093/bioinformatics/btaf251

**Published:** 2025-07-15

**Authors:** Shay Golan, Ido Tziony, Matan Kraus, Yaron Orenstein, Arseny Shur

**Affiliations:** Department of Computer Science, University of Haifa, Haifa 3498838, Israel; Efi Arazi School of Computer Science, Reichman University, Herzliya 4610101, Israel; Department of Computer Science, Bar-Ilan University, Ramat Gan 5290002, Israel; Department of Computer Science, Bar-Ilan University, Ramat Gan 5290002, Israel; Department of Computer Science, Bar-Ilan University, Ramat Gan 5290002, Israel; The Mina and Everard Goodman Faculty of Life Sciences, Bar-Ilan University, Ramat Gan 5290002, Israel; Department of Computer Science, Bar-Ilan University, Ramat Gan 5290002, Israel

## Abstract

**Motivation:**

Minimizers are the most popular *k*-mer selection scheme in algorithms and data structures analyzing high-throughput sequencing (HTS) data. In a minimizer scheme, the smallest *k*-mer by some predefined order is selected as the representative of a sequence window containing *w* consecutive *k*-mers, which results in overlapping windows often selecting the same *k*-mer. Minimizers that achieve the lowest frequency of selected *k*-mers over a random DNA sequence, termed the expected density, are desired for improved performance of HTS analyses. Yet, no method to date exists to generate minimizers that achieve minimum expected density. Moreover, for *k* and *w* values used by common HTS algorithms and data structures, there is a gap between densities achieved by existing selection schemes and the theoretical lower bound.

**Results:**

We developed GreedyMini, a toolkit of methods to generate minimizers with low expected or particular density, to improve minimizers, to extend minimizers to larger alphabets, *k*, and *w*, and to measure the expected density of a given minimizer efficiently. We demonstrate over various combinations of *k* and *w* values, including those of popular HTS methods, that GreedyMini can generate DNA minimizers that achieve expected densities very close to the lower bound, and both expected and particular densities much lower compared to existing selection schemes. Moreover, we show that GreedyMini’s *k*-mer rank-retrieval time is comparable to common *k*-mer hash functions. We expect GreedyMini to improve the performance of many HTS algorithms and data structures and advance the research of *k*-mer selection schemes.

**Availability and implementation:**

The toolkit, its source code, and precomputed minimizers for a variety of (k,w) pairs are available via https://github.com/OrensteinLab/GreedyMini.

## 1 Introduction

Minimizers are used in many bioinformatic applications, including sequence alignment, genome assembly, and data compression (Zheng *et al.* 2023, Moeckel *et al.* 2024, Ndiaye *et al.* 2024). Minimizers are schemes that select representative sets of *k*-mers from sequences so that among every *w* consecutive *k*-mers the smallest *k*-mer according to some predefined order is selected. By efficiently identifying and indexing these *k*-mers, the computational complexity and memory requirements associated with processing large genomic datasets can be significantly reduced.

Minimizers are commonly evaluated by their expected or particular density. The expected density is the expected frequency of selected *k*-mers within a random DNA sequence, while the particular density is the frequency of selected *k*-mers in a specific DNA sequence (Zheng *et al.* 2023). Low density indicates a sparse and efficient selection, which enhances the performance of high-throughput sequencing (HTS) algorithms and data structures by reducing their runtime and memory usage. Thus, minimizers with low density are desired.

Several methods were developed to generate orders leading to low-density minimizers. Traditional lexicographical order often results in high density (Roberts *et al.* 2004, Marçais *et al.* 2017). Universal hitting sets (UHSs) were introduced to design orders for low-density minimizers. But, expensive heuristics are required to generate UHS-based orders limiting them to k≤15 (Orenstein *et al.* 2016, 2017, Ekim *et al.* 2020). Decycling-based minimizers can be applied to any *k* value and achieve the lowest expected density compared to existing selection schemes to date (Pellow *et al.* 2023). Frequency-based orders can be used as a simple technique to generate minimizers with low particular density (Chikhi *et al.* 2015). More recently, DeepMinimizer was developed to utilize machine learning to distribute selected *k*-mers more evenly, directly targeting low particular density (Hoang *et al.* 2022). To date, no method solves the problem of generating a minimum-expected-density minimizers scheme, and there is a gap between the theoretical lower bound and the densities achieved by existing selection schemes (Kille *et al.* 2024). Some minimizers, such as Miniception (Zheng *et al.* 2020) and syncmers (Edgar 2021), and other *k*-mer selection schemes, such as mod-minimizer (Groot Koerkamp and Pibiri 2024) and strobemers (Sahlin 2021), employ “internal” minimizers and can benefit from the use of low-density minimizers.

Here, we present GreedyMini, a toolkit of novel methods to generate minimizers with low expected or particular density, to locally improve a given minimizer, to extend minimizers to larger alphabets, *k*, and *w*, and to measure the expected density of a given minimizer efficiently. We prove density guarantees for GreedyMini’s extensions to larger alphabets, and larger *k*. We combine these innovations into several pipelines to generate low-density DNA minimizers. We demonstrate over various combinations of *k* and *w* values, which are most practical for HTS analyses, that the pipelines generate minimizers that achieve lower density compared to existing selection schemes and very close to the theoretical lower bound (Kille *et al.* 2024) (e.g. within 1% for w=k−1) and at comparable *k*-mer rank-retrieval time as common *k*-mer hash functions. We expect GreedyMini to improve the runtime and memory usage of HTS algorithms and data structures and to lead to further advancements in generating minimizers and *k*-mer selection schemes with low density.

## 2 Preliminaries

For integers i≥0 and j≥i, we denote [i,j)={i,…,j−1}. Let Σ=[0,σ) be an alphabet and $S\in \Sigma^{+}$ be a nonempty string over Σ. *S*[*i*] denotes the letter at position *i* (the leftmost letter is *S*[0]), and S[i,j) refers to the substring covering the interval [i,j) of positions, so that S=S[0,|S|). Substrings of the form S[0,j) and S[i,|S|) are *prefixes* and *suffixes* of *S*, respectively. *Selection schemes* (defined below) operate by selecting substrings of small length *k*, called *k-mers*, in longer substrings called *windows*. We write *n-window* (and *n*-prefix, *n*-suffix, and *n*-string) to indicate length. Our algorithms assume the common unit-cost word-RAM model. If any integer function g(σ,k,w) appears as the time or space complexity of an algorithm, its values are assumed to fit into O(1) machine words; σ is treated as a constant. For a Boolean expression *B*, [*B*] equals 1 if *B* is true and 0 otherwise.

A *local* (*selection*) *scheme* with parameters (σ,k,w) is a map $f:\Sigma^{w+k-1}\to [0,w)$. This map acts on strings over Σ, selecting one position in each (w+k−1)-window so that in a window v=S[i,i+w+k−1) the position i+f(v) in *S* is selected.

The most popular metric to evaluate a local scheme by is the density of selected positions. Let f(S) denote the set of positions selected in a string *S* by a local scheme *f*. The *particular density of f on S* is $d_{f}(S)=\left|f(S)\right|/S|-k+1$. The *expected density of f* is the limit $d_{f}=\underset{n\to \infty }{\lim}\frac{1}{\sigma^{n}}\sum_{S\in \Sigma^{n}}d_{f}(S)$. The *density factor* of *f* is a normalization of $d_{f}$ by a factor of (w+1), i.e. $df_{f}=(w+1)d_{f}$.

Let π be a permutation (=a linear order) of the set of all σ-ary *k*-mers. We view π as a bijection $\pi :[0,\sigma^{k})\to \Sigma^{k}$ and say that a *k*-mer *u* has *rank i*, denoted by $\mathrm{ran}k_{\pi }(u)=i$, if π(i)=u. The *minimizer* (π,w) is a local scheme that maps each (w+k−1)-window to the starting position of its minimum-rank *k*-mer with ties broken to the left.

A subset $H\subseteq \Sigma^{k}$ is a UHS for *w* if every (w+k−1)-window contains at least one *k*-mer from *H*. For example, {00,01,11} is a UHS for σ=k=2 and any w>1. Let π be a linear order on $\Sigma^{k}$ and let *i* be such that $H=\{u|\mathrm{ran}k_{\pi }(u)<i\}$ is a UHS for *w*. Every permutation $\pi^{\prime }$ such that $\pi^{\prime }(j)=\pi (j)$ for all j∈[0,i) generates the same minimizer: $\left(\pi^{\prime },w)=(\pi ,w\right)$. Accordingly, we define a *UHS order on* $\Sigma^{k}$ (for *w*) to be any bijection ρ:[0,|H|)→H, where $H\subseteq \Sigma^{k}$ is a UHS for *w*. Any completion of ρ to a permutation π of $\Sigma^{k}$ defines the same minimizer, which we denote by (ρ,w). We define $\mathrm{ran}k_{\rho }(u)$ as above if u∈H and set $\mathrm{ran}k_{\rho }(u)=\infty$ otherwise. Since $\Sigma^{k}$ is a UHS for any *w*, a permutation of $\Sigma^{k}$ is a special case of a UHS order. Prior studies focused on generating small UHSs (Orenstein *et al.* 2017, DeBlasio *et al.* 2019).

For a given minimizer f=(ρ,w), a (w+k)-window *v* (which contains two overlapping consecutive (w+k−1)-windows and often termed *context*) is *charged* if its minimum-rank *k*-mer is either its prefix or its *unique suffix* (i.e. the *k*-suffix of *v* having no other occurrence in *v*); otherwise, *v* is *free*. An important observation is that every string *S* contains exactly |f(S)|−1 (not necessarily distinct) charged (w+k)-windows (Lemma 6 in Zheng *et al.* 2023). Since all possible *n*-strings have, in total, the same number of occurrences of each (w+k)-window, the expected density $d_{f}$ of a minimizer equals the fraction of charged windows in $\Sigma^{w+k}$ (Marçais *et al.* 2017, Zheng *et al.* 2020).

## 3 Methods

### 3.1 Generating minimizer orders greedily

We present a novel randomized algorithm GreedyE (Algorithm 1, E stands for expected) to generate low-density minimizers. Given parameters (σ,k,w), GreedyE generates a UHS order ρ on $\Sigma^{k}$ for *w*, assigning ranks to *k*-mers one by one, starting from rank 0 and stopping when the *k*-mers with assigned ranks constitute a UHS. GreedyE maintains a score function for all unranked *k*-mers. At each iteration, GreedyE creates a pool of low-scored *k*-mers, chooses a random *k*-mer from it, assigns the next rank to the chosen *k*-mer, and updates the scores of all unranked *k*-mers.

During an iteration, a (w+k)-window is *live* if all its *k*-mers are unranked. For an unranked *k*-mer *u*, let $X_{u}$ be the set of all live windows containing *u*, and let $Y_{u}\subseteq X_{u}$ consist of live windows containing *u* as a prefix or as a unique suffix. Then, $\mathrm{score}(u)=|Y_{u}|/|X_{u}|$. The intuition behind selecting a low-scored *k*-mer is the following: choosing *u* to have the next rank clarifies the status (free or charged) of the windows from $X_{u}$ and only of them, and those from $Y_{u}$ are charged. Thus, score(u) locally approximates the fraction of charged windows among all windows, which is the fraction we want to minimize.

Algorithm 1: GreedyE: generating a low-density UHS order

**Require:** alphabet size σ, *k*-mer length *k*, window size *w*

**Ensure:** UHS order ρ for *w* on $\Sigma^{k}$

1: rank←[∞,…,∞]▹ initialize ranks of all *k*-mers

2: X,Y←  init_counts(w,k,σ)▹  $X[u]=|X_{u}|,Y[u]=|Y_{u}|$

3: for each *k*-mer *u*

4: **for**  r←0 to $\sigma^{k}-1$  **do**▹ current rank to be assigned

5:   u←select_candidate(X,Y)▹ select *k*-mer *u* among

6:          the *k*-mers with (almost) lowest $\left|Y_{u}|/|X_{u}\right|$

7:   **if**  u=null  **then**▹ no live windows, X=[0,…,0]

8:   **return**  rank▹  ρ is defined by the ranks of *k*-mers

9:   **end if**

10:     X,Y←update_counts(X,Y,u,rank)▹ for next iteration

11:     rank[u]←r▹ assign rank *r* to the selected *k*-mer *u*

12: **end for**

In our implementation, we define low-scored *k*-mers as follows. For each run of GreedyE, we sample a parameter α from a user-defined range: $\alpha ∼\text{LogUniform}(\alpha_{\min},\alpha_{\max})$. At each iteration of GreedyE, an unranked *k*-mer *u* is considered low-score if its *inverted score* (X[u]−Y[u])/Y[u] is at least αI, where *I* is the maximum inverted score at this iteration. With $\alpha_{\min}=\alpha_{\max}=1$, GreedyE selects a lowest-score *k*-mer at each iteration but still uses randomization when several *k*-mers are tied for the lowest score. Decreasing $\alpha_{\min}$ and $\alpha_{\max}$ expands the search space of low-density minimizers at no additional space or runtime costs, and thus has the potential to improve the best result over multiple runs.

The natural computational requirements for implementing GreedyE are $\Omega (\sigma^{k})$ space to store the order, $\Omega (\sigma^{k})$ time per iteration to select a low-score *k*-mer, and $\Omega (w\sigma^{k+w})$ time to scan each window once. Theorem 1 presents our implementation, which meets all these lower bounds up to an additional O(w) space. We prove the theorem and give the pseudocode of nontrivial auxiliary functions in Supplementary Section S4. Since GreedyE requires exponential time and space, it can be used to generate minimizers for practical (k,w) pairs only over the binary alphabet. In Section 3.3, we define the extension of minimizers to larger alphabets with density guarantees, thus making GreedyE practical for common HTS applications.

Theorem 1.
GreedyE *can be implemented to run in* $O(\sigma^{k}+w)$  *space and* $O(|H|(\sigma^{k}+w\sigma^{w}))$  *time, where H is the constructed UHS.*

Remark 1.

- *For every UHS H*, $\sigma^{k}/k<|H|\leq \sigma^{k}$*. In our experiments, the overall average size of a UHS generated by* GreedyE  *was <*$3\sigma^{k}/k$*. Examples of UHS size distribution are presented in Supplementary Fig. S2.*
-
GreedyE *can be implemented with the running time* $O(|H|w\sigma^{2k})$*, which is linear in w but has heavier dependence on k. We found no practical use for this implementation, so we only briefly describe it in Supplementary Section S4.*

To generate minimizers of low *particular* density with respect to a specific input string *S*, we present an analog of GreedyE, called GreedyP. In this case, each window has a weight equal to the number of its occurrences in *S*. GreedyP computes and stores the windows occurring in *S* with their weights at an additional expense of O(|S|) time and $O(\min\{|S|,\sigma^{w+k}\})$ space, since the number of (w+k)-windows of positive weight is bounded by the observed windows in *S* and by $\sigma^{w+k}$. Then, the only difference with GreedyE (Algorithm 1) is that the arrays X and Y contain *sums of weights* of windows rather than the numbers of windows. For the case where $|S|\ll \sigma^{w+k}$, GreedyP can be implemented more efficiently based on a linked list of windows with positive weight.

### 3.2 Improving minimizers by hill climbing

We apply a randomized version of the standard local search technique called *hill climbing* to improve a given minimizer (ρ,w), where ρ is a UHS order for *w*. At each iteration, the search algorithm samples a random rank *r* such that both *r* and r+1 are assigned in ρ and calls the function Swap(ρ,w,r), defined as follows. Swap(ρ,w,r) generates the UHS order $\rho^{\prime }$ from ρ by swapping the *k*-mers with ranks *r* and r+1 and calculates $\Delta_{\rho ,\rho^{\prime }}≜d_{\left(\rho ,w\right)}-d_{\left(\rho^{\prime },w\right)}$. If $\Delta_{\rho ,\rho^{\prime }}>0$, Swap replaces ρ with $\rho^{\prime }$; if $\Delta_{\rho ,\rho^{\prime }}<0$, Swap does nothing; if $\Delta_{\rho ,\rho^{\prime }}=0$, Swap replaces ρ with $\rho^{\prime }$ with *P*=.5. The search stops when a user-defined time limit or number of iterations is reached.

Theorem 2 presents the complexity of our two efficient implementations of Swap. Our default implementation (i) uses recursive depth-first traversal (SwapDFS), while (ii) is designed for the case of large *w* and based on dynamic programming (SwapDP). The proof of Theorem 2 and the pseudocodes of SwapDFS and SwapDP are presented in Supplementary Section S3.

Theorem 2. *Let* ρ  *be a UHS order on* $\Sigma^{k}$  *for w, and let the ranks* r,r+1  *be assigned in* ρ*. The function* Swap(ρ,w,r)  *can be implemented in either (i)* $O(\sigma^{k}+\sigma^{w})$  *time and* O(w)  *additional space or (ii)* $O(w\sigma^{k})$  *time and* $O(\sigma^{k})$  *additional space.*

### 3.3 Extending orders to larger σ and *k*

A local scheme *f* with the parameters (σ,w,k) can be extended to a local scheme $f^{\prime }$ with the parameters $\left(\sigma ,w,k^{\prime }\right)$, where $k^{\prime }>k$, by a simple rule (Marçais *et al.* 2018): in every $\left(w+k^{\prime }-1\right)$-window, $f^{\prime }$ picks the position chosen by *f* in the (w+k−1)-prefix of this window. Trivially, $d_{f}=d_{f^{\prime }}$. In a similar way, a local scheme $f_{\gamma }$ with the parameters (σγ,w,k) can be built from *f*. Namely, the alphabet is partitioned into σ classes, and in every window *v* $f_{\gamma }$ picks the position chosen by *f* in the window $\bar{v}$, obtained from *v* by replacing each letter with its class. Here as well, $d_{f}=d_{f_{\gamma }}$.

Such extensions may suffice for practice. But, their main theoretical limitation is that $f^{\prime }$ and $f_{\gamma }$ do not inherit the property “to be a minimizer” from *f*. Namely, if *f* is a minimizer, then $f_{\gamma }$ is never a minimizer, while the status of $f^{\prime }$ depends on *f* and *w*. In particular, if $w>k^{\prime }$, then $f^{\prime }$ is not a minimizer for any *f*. See Supplementary Section S5 for a detailed discussion, including the earlier attempt (DeBlasio *et al.* 2019) of a minimizer-to-minimizer extension.

**Figure 1.**
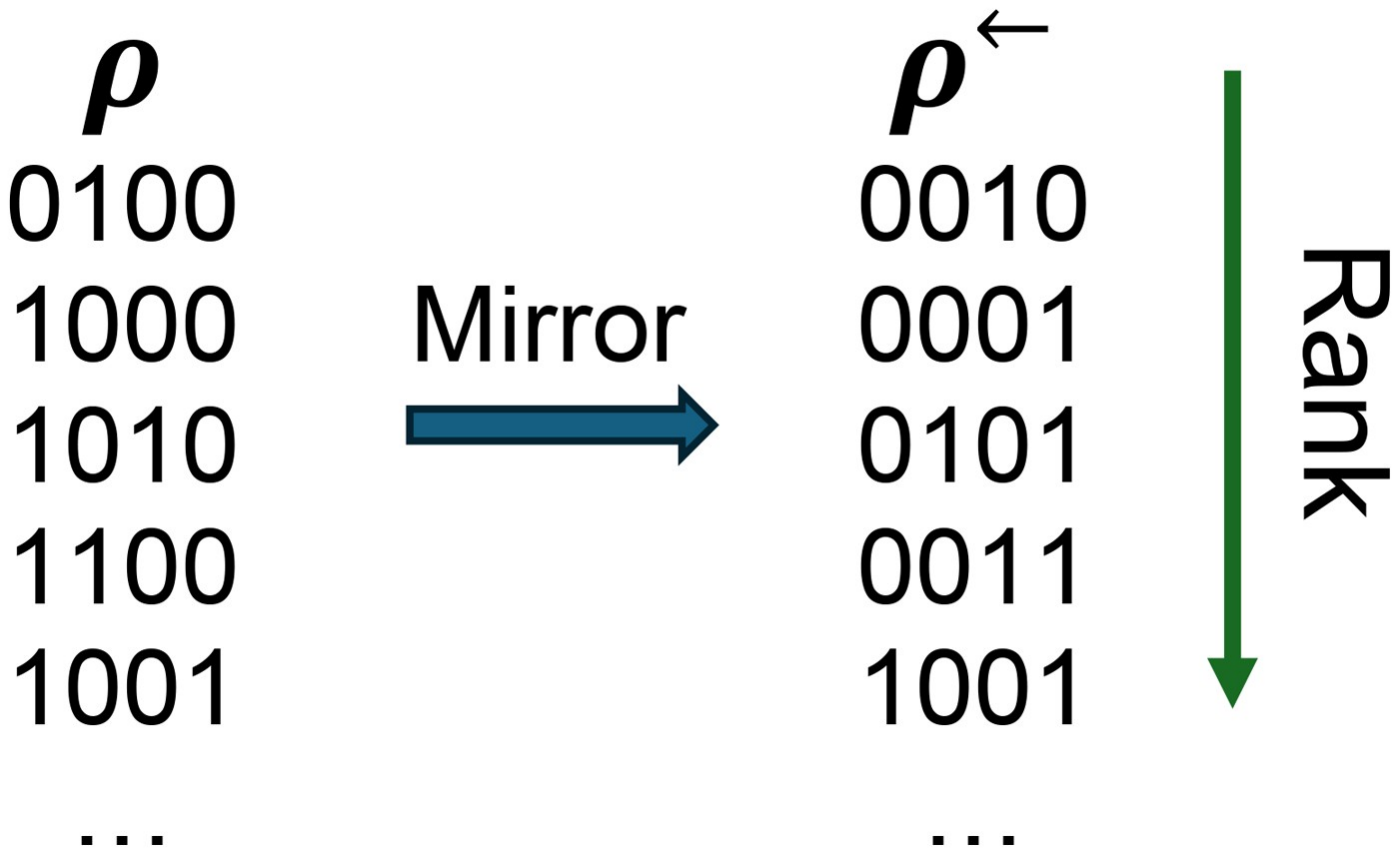
An example of a binary order ρ on 4-mers and its mirror $\rho^{\overset{{}_{\leftarrow }}{}}$.

We upgrade these trivial extensions to obtain minimizer-to-minimizer extensions, and prove their density guarantees. We define *dual minimizers* as local schemes that act like minimizers but break ties to the right. For a minimizer *f*, we denote the dual minimizer using the same order as *f* by $f^{\overset{{}_{\leftarrow }}{}}$. Notice that $d_{f}$ and $d_{f^{\overset{{}_{\leftarrow }}{}}}$ are close: only the windows containing their minimum-rank *k*-mer as a nonunique prefix or a nonunique suffix are charged differently (e.g. the average difference in expected density was <0.15% over 1000 random minimizers for σ=2, k=8, and w=12). Given a UHS order ρ for *w* on $\Sigma^{k}$, we define its *mirror* as the UHS order $\rho^{\overset{{}_{\leftarrow }}{}}$ such that $\mathrm{ran}k_{\rho^{\overset{{}_{\leftarrow }}{}}}(u)=\mathrm{ran}k_{\rho }(\overset{{}_{\leftarrow }}{u})$ for all $u\in \Sigma^{k}$, where $\overset{{}_{\leftarrow }}{u}$ is *u* reversed (Fig. 1). Lemma 1 connects mirrors to dual minimizers.

Lemma 1. *If* ρ  *is a UHS order for w, then* $d_{\left(\rho^{\leftarrow },w\right)}=d_{{\left(\rho ,w\right)}^{\leftarrow }}$.

Proof.

If a (w+k)-window *v* is charged by the scheme ${\left(\rho ,w\right)}^{\overset{{}_{\leftarrow }}{}}$, its *k*-mer *u* with the minimum ρ-rank is either a suffix or a unique prefix of *v*. Hence, in the window $\overset{{}_{\leftarrow }}{v}$ the *k*-mer $\overset{{}_{\leftarrow }}{u}$ has the minimum $\left(\rho^{\overset{{}_{\leftarrow }}{}}\right)$-rank and is either a prefix or a unique suffix; respectively, $\overset{{}_{\leftarrow }}{v}$ is charged by $\left(\rho^{\overset{{}_{\leftarrow }}{}},w\right)$. As all steps are reversible, we get a bijection between the sets of charged windows of the two schemes. □

We transform an arbitrary minimizer *f* with the parameters (σ,k,w) to a minimizer $f^{\prime }$ with the parameters (σγ,k,w) for an integer γ>1 as follows. Let Γ=[0,γ) and consider the alphabet $\Sigma \times \Gamma =\{\left[\begin{array}{c} a\\ b \end{array}\right]|a\in \Sigma ,b\in \Gamma \}$ of size σγ. Every string $S=\left[\begin{array}{c} a_{0}\\ b_{0} \end{array}\right]\left[\begin{array}{c} a_{1}\\ b_{1} \end{array}\right]\cdots \left[\begin{array}{c} a_{n-1}\\ b_{n-1} \end{array}\right]\in {\left(\Sigma \times \Gamma \right)}^{n}$ has two *projections* $S_{\Sigma }=a_{0}\cdots a_{n-1}\in \Sigma^{n}$ and $S_{\Gamma }=b_{0}\cdots b_{n-1}\in \Gamma^{n}$. Given a UHS order ρ for *w* on $\Sigma^{k}$ and a linear order τ on $\Gamma^{k}$, we define a UHS order ρ×τ for *w* on ${\left(\Sigma \times \Gamma \right)}^{k}$ by setting $\mathrm{ran}k_{\rho \times \tau }(u)=\gamma^{k}\mathrm{ran}k_{\rho }(u_{\Sigma })+\mathrm{ran}k_{\tau }(u_{\Gamma })$; in particular, $\mathrm{ran}k_{\rho \times \tau }(u)=\infty$ iff $\mathrm{ran}k_{\rho }(u_{\Sigma })=\infty$. The following theorem presents a minimum density guarantee over the minimizers (ρ×τ,w) and $\left(\rho^{\leftarrow }\times \tau ,w\right)$. Its proof is provided in Supplementary Section S6.

Theorem 3. *Let* ρ  *be a UHS order on* $\Sigma^{k}$  *for w and* τ  *be a linear order on* $\Gamma^{k}$. *Then*, $\min\{d_{\left(\rho \times \tau ,w\right)},d_{\left(\rho^{\leftarrow }\times \tau ,w\right)}\}\leq (d_{\left(\rho ,w\right)}+d_{{\left(\rho ,w\right)}^{\leftarrow }})/2$.

We use a similar approach to obtain the same density guarantee for the extension of a minimizer with parameters (σ,k,w) to a minimizer with the parameters (σ,k+1,w). Given a UHS order ρ on $\Sigma^{k}$ for *w*, we define its *extensions* $\rho_{1}$ and $\rho_{2}$ on $\Sigma^{k+1}$ as follows. For $u\in \Sigma^{k}$ and a∈Σ, we set $\mathrm{ran}k_{\rho_{1}}(ua)=\sigma \cdot \mathrm{ran}k_{\rho }(u)+a$ and $\mathrm{ran}k_{\rho_{2}}(au)=\sigma \cdot \mathrm{ran}k_{\rho^{\overset{{}_{\leftarrow }}{}}}(u)+\sigma -a-1$. We prove the next theorem in Supplementary Section S7.

Theorem 4. *Let* ρ  *be a UHS order on* $\Sigma^{k}$  *for w, and let* $\rho_{1}$  *and* $\rho_{2}$  *be its extensions. Then*, $\min\{d_{\left(\rho_{1},w\right)},d_{\left(\rho_{2},w\right)}\}\leq (d_{\left(\rho ,w\right)}+d_{{\left(\rho ,w\right)}^{\leftarrow }})/2$.

### 3.4 Measuring the density of a given minimizer

A standard approach to measure the expected density of a given selection scheme is to generate a long (pseudo)random string and count sampled positions. However, the exact calculation of the expected density is preferred whenever it is feasible. As was pointed out by Marçais *et al.* (2017), the exact density can be computed by processing a de Bruijn sequence of order w+k instead of a random string, as such a sequence contains each (w+k)-window exactly once. In this way, the expected density can be computed in $O(\sigma^{w+k})$ time and O(w+k) space using a fast shift rule for de Bruijn sequences (Fredricksen and Maiorana 1978, Sawada *et al.* 2017).

We present two new algorithms that calculate the exact expected density of a given minimizer more efficiently. The recursive algorithm DenDFS processes all (k+w)-windows as a group of trees, which results in an efficient reuse of computed information compared to the sequential processing of a de Bruijn sequence. The algorithm DenDP computes dynamic-programming tables over *k*-mers and runs in time linear in *w* at the expense of $\Theta (\sigma^{k})$ space. The complexity of DenDFS and DenDP is given by Theorem 5(i) and (ii), respectively. The algorithms and the proof of Theorem 5 are presented in Supplementary Section S2.

Theorem 5. *Let* ρ  *be a UHS order on* $\Sigma^{k}$  *for w and let H be its UHS. The expected density of the minimizer* (ρ,w)  *can be computed in either (i)* $O(|H|\sigma^{w})$  *time and* O(w)  *space or (ii)* $O(w|H|\sigma^{k})$  *time and* $O(\sigma^{k})$  *space.*

Remark 2. *Small UHS size, typically at the output of* GreedyE  *(Supplementary Fig. S2), contributes to the advantage of* DenDFS  *over the processing of a de Bruijn sequence. However*, DenDFS  *is still preferable to processing a de Bruijn sequence even if no UHS is known for the input order, as we demonstrate in Supplementary Section S9.*

### 3.5 GreedyMini toolkit and pipelines

We combined the novel techniques from Sections 3.1–3.4 into a toolkit called GreedyMini. Its primary purpose is the generation of low-density DNA minimizers for given parameters *k* and *w*.

The main tools are GreedyE and GreedyP (Section 3.1), implemented for the *binary* alphabet. GreedyE takes the parameters *k*, *w*, a range $\left(\alpha_{\min},\alpha_{\max}\right)$, and returns a UHS order ρ on ${\left\{0,1\right\}}^{k}$ for *w* such that the minimizer (ρ,w) has low expected density. GreedyP has the same input parameters plus a sequence *S*, and outputs an *S*-specific analog of a UHS order. Namely, ρ:[0,|H|)→H ranks a set $H\subseteq {\left\{0,1\right\}}^{k}$ such that every (w+k−1)-window in *S* contains a *k*-mer from *H*. The minimizer (ρ,w) has low particular density for *S*.

The tools SwapDFS and SwapDP (Section 3.2) take the parameter *w* and a UHS order ρ on ${\left\{0,1\right\}}^{k}$ for *w* as input and return an order $\rho^{\prime }$ on ${\left\{0,1\right\}}^{k}$ such that $d_{\left(\rho^{\prime },w\right)}<d_{\left(\rho ,w\right)}$; if no such order $\rho^{\prime }$ was found, ρ is returned. By default, SwapDFS is used, while SwapDP is intended to cover large values of *w*.

The tools DenDFS and DenDP (Section 3.4) compute the exact expected density of *binary* minimizers with DenDFS being the default method and DenDP covering large values of *w*.

Finally, the toolkit includes auxiliary functions described in Section 3.3. Mirror receives a binary UHS order ρ and returns the order $\rho^{\overset{{}_{\leftarrow }}{}}$; Extend-k receives a binary minimizer and extends it to a larger value of *k*; Extend-σ receives a binary minimizer and extends it to the DNA alphabet (taking σ=γ=2 in the extension definition, with τ being the lexicographic order).

We implemented several pipelines as part of GreedyMini.

#### 3.5.1 GM-expected: generating a DNA minimizer for (k,w)

To construct a DNA minimizer with low *expected* density for given *k* and *w*, we proceed in three stages (Supplementary Fig. S4A).

Generate: Given *k* and *w*, run multiple instances (by default 4096) of GreedyE(2,k,w); the default range $\left(\alpha_{\min},\alpha_{\max}\right)$ to sample the parameter α is (0.94,0.9996). Report a lowest density minimizer or its mirror, whichever has lower density.

Improve: For the minimizer reported by stage Generate, run an instance of SwapDFS on each available CPU core (64 on our machine) with the time limit set to the runtime of stage Generate (in our experiments, this resulted in $>400\cdot 2^{k}$  Swap calls per core). Report a lowest density minimizer.

Extend: Given the minimizer (ρ,w) from stage Improve, report the DNA minimizer Extend-σ(ρ,w) or $\mathrm{Extend}-\sigma (\rho^{\overset{{}_{\leftarrow }}{}},w)$, whichever achieves lower density.

#### 3.5.2 GM-particular: generating a DNA minimizer for (k,w) and sequence *S*

To construct a DNA minimizer with low *particular* density for given *k*, *w*, and a sequence *S*, we perform stage Generate, replacing GreedyE by GreedyP, followed by stage Extend to obtain the result (Supplementary Fig. S4B). We skip stage Improve since we do not have an efficient implementation of Swap (comparable to Theorem 2) that can process the list of (k+w)-windows of *S*.

#### 3.5.3 GM-improve: generating a DNA minimizer for a large *w*

If running GM-expected on the pair (k,w) is expected to take too much time due to a large *w*, we start with a binary minimizer obtained for the pair $\left(k,w^{\prime }\right)$ for some $w^{\prime }<w$. We run an instance of SwapDP on each available CPU core within a manually set time limit (10 h by default) and choose the lowest density minimizer over these runs. For the chosen minimizer, we run stage Extend to obtain the final result (Supplementary Fig. S4C). Additionally, GM-improve can be used to improve any binary minimizer via the use of SwapDP.

#### 3.5.4 GM-k: generating a DNA minimizer with increased *k*

If running GM-expected on the pair (k,w) resulted in a minimizer having higher density than the minimizer obtained for the pair (k−1,w), we start with the binary minimizer obtained for (k−1,w), apply Extend-k on it, and run stage Improve within a manually set time limit. Then, we run stage Extend to obtain the result. This pipeline is useful for the case where *k* is large enough and *w* is small enough, as running Swap takes just $O(2^{w})$ time, while the runtime of an instance of GreedyE is $O(2^{2k})$.

#### 3.5.5 Trivial extensions

If running SwapDP and SwapDFS for the pair (k,w) takes too much time or is even infeasible, we use trivial extensions. For a trivial *k*-extension, we take a binary minimizer for some parameters $k^{\prime }<k$ and *w*, apply Extend-k, and run stage Extend on it. For a trivial *w*-extension, we take a binary order ρ obtained for some parameters *k* and $w^{\prime }<w$, and run stage Extend on the minimizer (ρ,w).

## 4 Results

### 4.1 Benchmarking *k*-mer selection schemes

We used GreedyMini to generate low-density DNA minimizers for various *k*, *w* combinations and benchmarked the results against the minimizers constructed by the state-of-the-art methods: double decycling (Pellow *et al.* 2023), miniception (Zheng *et al.* 2020), and the recent open-closed syncmer (Koerkamp *et al.* 2024), which mixes the ideas of the previous two. For these methods, we used the implementations from the mod-minimizer C${}^{++}$ GitHub repository (Groot Koerkamp and Pibiri 2024) and measured their density on a random 4-ary $10^{7}$-string. We excluded decycling (Pellow *et al.* 2023), DOCKS (Orenstein *et al.* 2017), and PASHA (Ekim *et al.* 2020) methods from the comparison as they were previously outperformed by double decycling (Pellow *et al.* 2023). We also compared to the lower bound of (Kille *et al.* 2024): $d_{f}\geq \max\{\frac{1}{w+k}\left\lceil \frac{w+k}{w}\right\rceil ,\frac{1}{w+k^{\prime }}\left\lceil \frac{w+k^{\prime }}{w}\right\rceil \}$, where $k^{\prime }=\left\lceil \frac{k-1}{w}\right\rceil w+1$. This bound is valid for all *forward* schemes, which constitute a wide class of local schemes including all minimizers.

To benchmark in the mode “fixed *w*, variable *k*,” we took w=8 and w=12. For w=8, we ran GM-expected for all k∈[3,18) and made a trivial *k*-extension up to k=30. For w=12, we ran GM-expected for all k∈[3,17) and extended trivially up to k=26. Similarly, to benchmark in the mode “fixed *k*, variable *w*,” we took k=8 and k=12. For k=8, we ran GM-expected for all w∈[3,20), and made a trivial *w*-extension up to w=100. For k=12, we ran GM-expected for all w∈[3,17), and then ran GM-improve subsequently for w=20,25,30, taking the previous result as the starting point. We then trivially extended the resulting minimizer up to w=100.

In addition, we ran GM-expected over all (k,w) pairs with 3≤k,w≤15 and focused on the mode “w≈k.” There were a few cases where the density obtained by GM-expected for (k,w) was higher than the one obtained for (k−1,w). In each such case, we ran GM-k to improve the result.

Finally, we addressed specific (k,w) pairs used in popular HTS methods: (9,16) (KMC 3) and (15,17) (Kraken) were processed by GM-expected; for (7,22) and (7,49) (both KMC 2), we made a trivial *w*-extension from w=15; for (21,11) (SSHash), (29,11) (Giraffe), and (31,5) (Kraken 2), we made a trivial *k*-extension from k=15; for (19,30) (GraphAligner) we made a trivial *k*-extension from k=12.

We computed the expected density of the resulting DNA minimizers in two steps. First, we computed the exact density of the corresponding binary minimizer, using DenDFS by default and DenDP for *w*-extensions, and then added the correction for the numbers of bad/good (k+w)-windows (Supplementary Section S6). We computed this correction exactly for k+w≤26 and estimated it from a sample of $10^{8}$ windows over Γ for 27≤k+w≤30. For k+w≥31 and for all trivial extensions, we report the upper bound by Theorem 3.

We also ran GM-particular to generate minimizers with low particular density for a DNA sequence of $10^{6}$ nucleotides from Chromosome X for w=12 and all k∈[3,17), and for k=8 and all w∈[3,20). We compared the results to the same set of benchmarked selection schemes; our attempts to compare to DeepMinimizer (Hoang *et al.* 2022) and polar sets (Zheng *et al.* 2021) failed, because their GitHub implementations raised errors and lacked documentation (Supplementary Section S11). We provide additional information (the minimizer construction runtime, maximum memory usage, and user recommendations) on GM-expected and GM-particular in Supplementary Section S12.

### 4.2 Expected and particular density results

**Figure 2.**
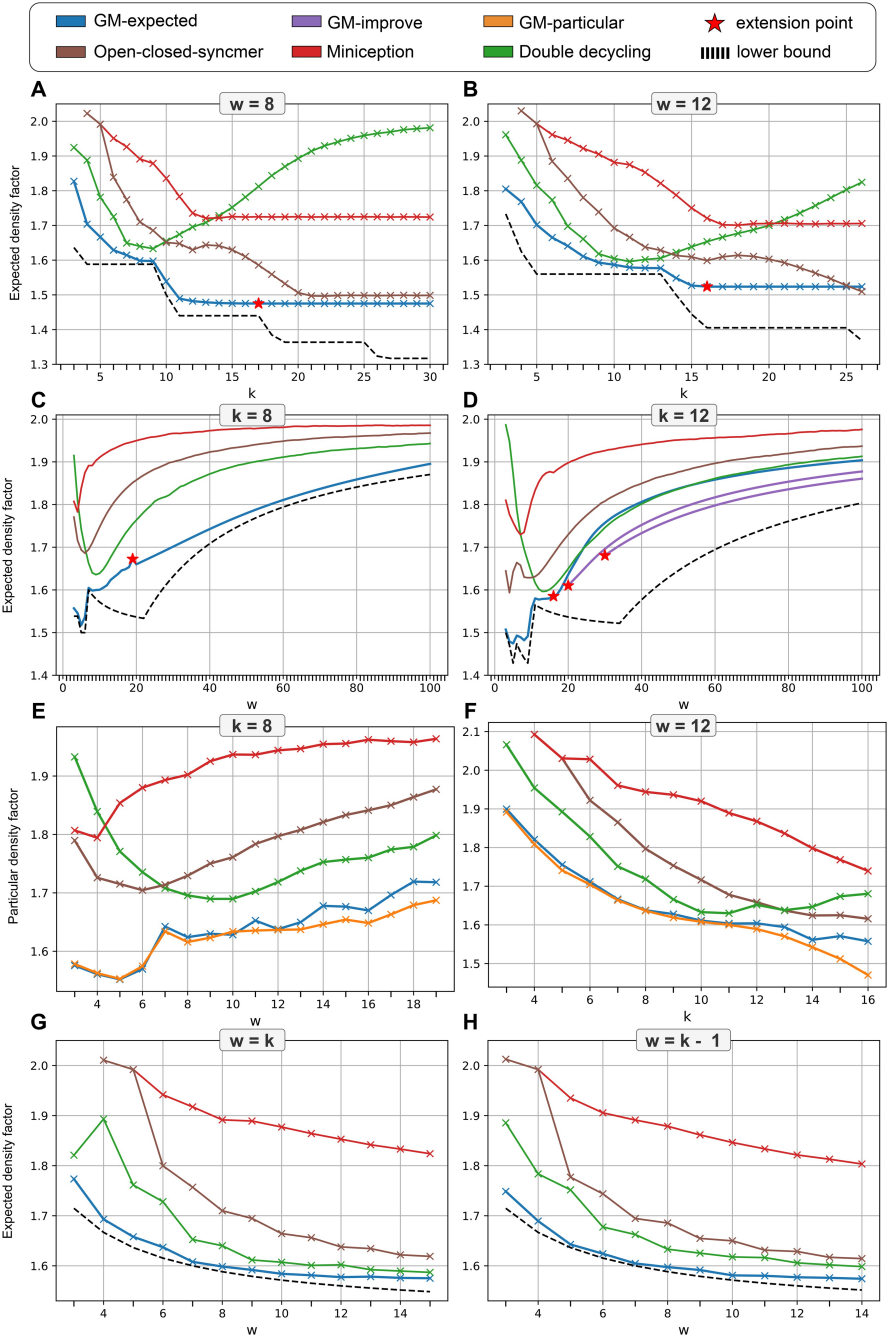
Performance of various DNA *k*-mer selection schemes over various (k,w) values. (A–D) Expected density comparisons over fixed *k* or *w*. (E, F) Particular density comparisons over 1 million nucleotides from Chromosome X. (G, H) Expected density comparisons over w∈{k,k−1}.

For fixed *w* (Fig. 2A and B), GM-expected outperformed all other minimizers, losing to open-closed syncmer in a single point (12,26). For k=8 (Fig. 2C), GM-expected outperformed all other minimizers by a large margin, even though it uses the trivial *w*-extension. For k=12 (Fig. 2D), trivial *w*-extension of the GM-expected from w=16 is not enough: its result is nearly tied with the result of double decycling for w>20. However, the use of GM-improve results in a clear gap of 2.40%–3.86%. We observed similar trends for particular density (Fig. 2E and F), where the best performers were GM-particular and GM-expected, with GM-particular achieving clearer superiority for w+k>24. Finally, in the case w∈{k,k−1} (Fig. 2G and H), GM-expected generated minimizers that have expected density within 1% of the lower bound (Kille *et al.* 2024).

### 4.3 Density comparisons for popular HTS methods

To emphasize the relevance and potential impact of GreedyMini, we calculated the expected density factors of minimizers generated by GM-expected over (k,w) combinations used by popular HTS methods (Wood and Salzberg 2014, Deorowicz *et al.* 2015, Li 2016, 2018, Kokot *et al.* 2017, Wood *et al.* 2019, Rautiainen and Marschall 2020, Andreace *et al.* 2021, Sirén *et al.* 2021, Pibiri 2022). We compared the results of GreedyMini to those of double decycling, miniception, open-closed syncmer, and random minimizer schemes.

**Figure 3.**
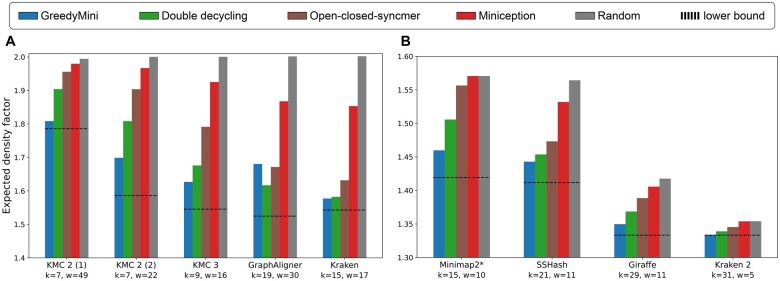
Density comparison over popular HTS methods. (A) Comparisons with other minimizers for k<w. (B) Comparison with other minimizers when plugged into a mod-minimizer scheme for k>w. *These (k,w) values also apply to Minimap (Li 2016) and MetaProb 2 (Andreace *et al.* 2021). We calculated the densities over $10^{7}$-long random DNA sequences.

For the combinations with w>k in four out of five cases, GreedyMini achieved the best result (Fig. 3A, Supplementary Table S1). It loses to double decycling and open-closed syncmer schemes only for the pair (19,30) used by GraphAligner. Among four considered combinations with k>w, GreedyMini shows the best result for three and second best (after open-closed syncmer) for the remaining one (Supplementary Table S1).

However, among all known forward schemes, the best results in the case k>w are achieved by the mod-minimizer scheme (Groot Koerkamp and Pibiri 2024). This scheme is not a minimizer, but uses a minimizer (for some $k^{\prime }<k$) as an intermediate step. Thus, we plugged the minimizers obtained by GreedyMini and all benchmarked methods into the mod-minimizer scheme, using the parameter r=4 as in the original setting (Koerkamp *et al.* 2024). We measured the density of obtained schemes over a $10^{7}$-long random DNA sequence. In all four cases, GreedyMini showed the best result (Fig. 3B, Supplementary Table S2), achieving a density <0.1% away from the lower bound for the parameters of Kraken 2.

### 4.4 *k*-mer rank-retrieval times

We next evaluated the sampling time for the DNA minimizers produced by GreedyMini. Sampling a string *S* consists of two tasks: retrieving the ranks of all *k*-mers and computing the minimum rank in a sliding window. The second task is independent of the minimizer used, so we focused on the rank-retrieval time.

We ran a code that assigns ranks to *k*-mers on a random DNA sequence of $3\times 10^{9}$ nucleotides, and calculated the average runtime over 10 runs for robust estimation. To simulate an order generated by GM-expected or GM-particular, we stored a binary order in memory and extended it by Extend-σ. For benchmarking, we used two ranking algorithms that do not require storing an order in memory: a trivial XOR-based hash function, which is used by some minimizers to create a pseudorandom order, and std::hash, which is the default hash implementation in C${}^{++}$. We ran our code on an AMD Ryzen 7 7800 × 3D (L1/L2/L3 cache size: 512KB/8MB/96MB) alongside 32GB of DDR5 RAM.

**Figure 4.**
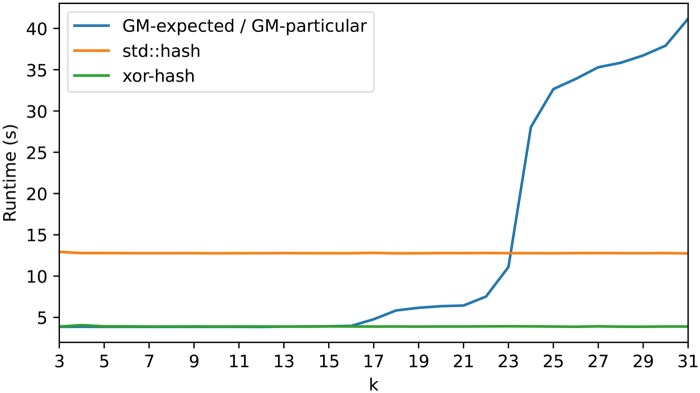
Runtime to assign ranks to *k*-mers over a $3\times 10^{9}$-long random DNA sequence, averaged over 10 runs.

For values for which GM-expected generates DNA minimizers in a reasonable runtime (i.e. k≤15), it is also very fast to retrieve *k*-mer ranks using the resulting minimizers (Fig. 4). The rank-retrieval times for GreedyMini match those for the XOR-based hash function (<4 s over 3 billion nucleotides including the generation of the random input string). For 17≤k≤22, there was a small increase in time, which still remains well below the results of std::hash. Only for k>22, the rank-retrieval time steeply increases, presumably switching to storing (some of) the order in the slowest cache or even in the main memory. Since any order generated by GreedyMini for k>22 is likely to use a trivial *k*-extension from some smaller $k^{\prime }$, then only the order of $k^{\prime }$-mers needs to be stored. Thus, on the tested hardware the sampling time of the minimizers generated by GreedyMini is guaranteed to be competitive.

### 4.5 Effect of individual steps on density results

We next measured and analyzed the impact of different steps in GreedyMini on the obtained densities. We tested the effect of transforming binary minimizers to DNA minimizers by Extend-σ and the effect of the local improvements by Swap.

The difference in expected density between the binary minimizers generated by stages Generate and Improve of GM-expected and the DNA minimizers obtained with Extend-σ in stage Extend was <1% for all minimizers generated over 5≤k≤15 and 3≤w≤15 (Supplementary Table S3); moreover, for k≥9 this difference was well below 0.1% in all cases. Thus, we conclude that the effect of Extend-σ on density is negligible.

The contribution of Swap was minor for most combinations (k,w) we tested (with the orders generated by GreedyE). For 3≤w≤15, k≤6, there was no reduction in expected density for most pairs (k,w), possibly due to a global or local optimum. For 3≤w≤15 and 7≤k≤15, the average reduction in expected density was 0.26%±0.15% with the reduction 0.67% for the pair (13,10) being the best improvement (Supplementary Table S4). In contrast, if the pair (k,w) is out of the range of GreedyE, the use of Swap in the GM-improve pipeline resulted in substantial improvements. For example, for the pair (12,30)  Swap has reduced the expected density by 4.35%.

Next, we tested how effective Swap is on other minimizer orders. For this test, we ran SwapDFS on a random binary order and a binary double decycling order for k=w=15. We compared the results to the density factor 1.576 of the GreedyE order selected by GM-expected. After $8\times 10^{7}$ calls to Swap (which took more time to run than the default number of GreedyE calls), the density factor of the random order dropped from 2 to only 1.952, while the density factor of the double decycling order improved from 1.608 to only 1.597 (Supplementary Fig. S7). This test demonstrates that Swap alone cannot bring other orders to the level of GreedyE.

Overall, we conclude that the principal role in constructing low-density minimizers is played by GreedyE/GreedyP, with mostly marginal effect of other steps on density.

### 4.6 Toward optimal minimizers

**Figure 5.**
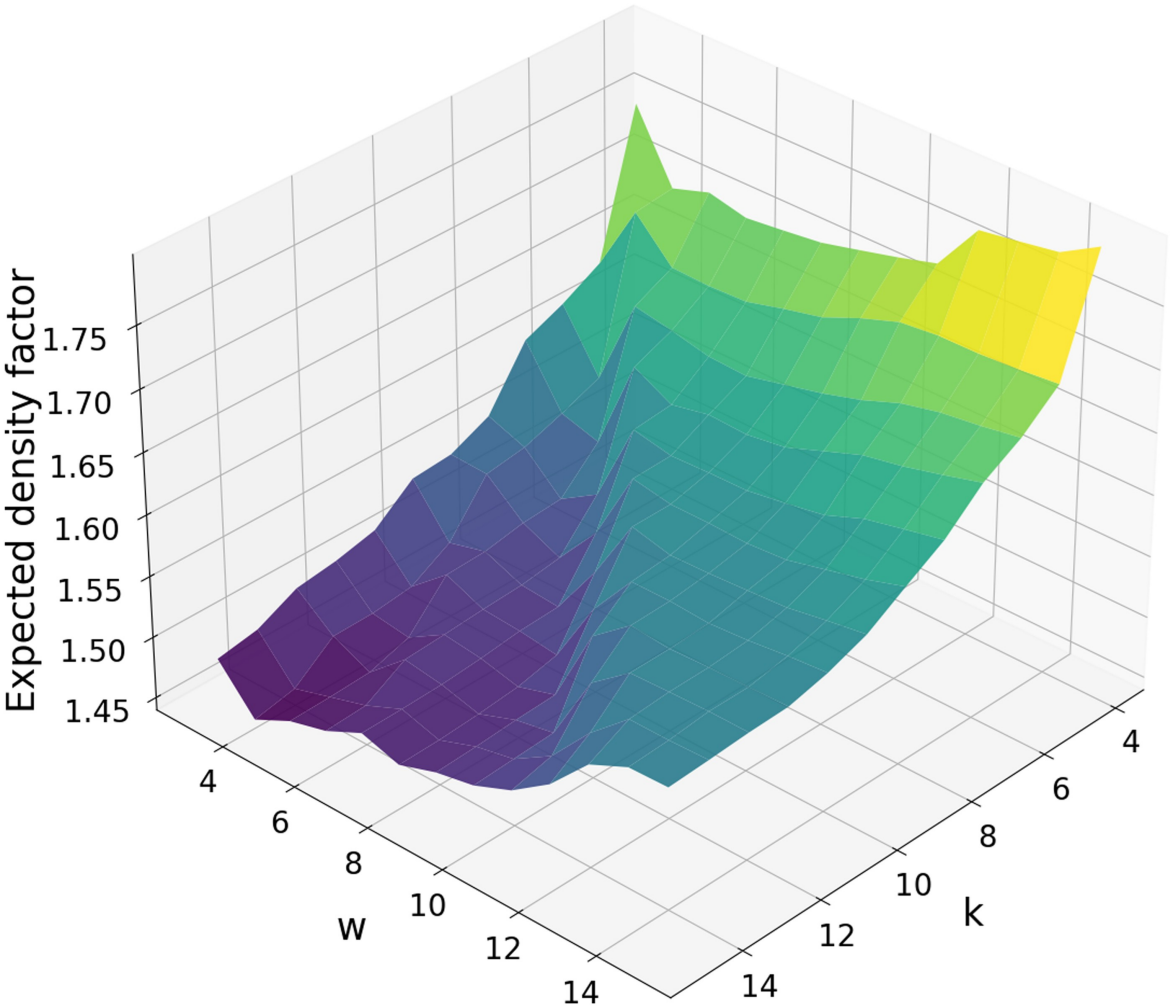
Density factors of the best binary minimizers generated by GreedyMini pipelines.

As already mentioned in Section 4.2, the densities of minimizers generated by GreedyMini pipelines are close to the lower bound on the density of forward schemes (Kille *et al.* 2024). Moreover, the *binary* minimizers by GreedyMini pipelines in the process hit this lower bound for the following (k,w) pairs: (4,3), (7,3), (10,3), (5,4), and (6,5). Apart from proving that, for some (k,w) combinations, minimizers are as good as general forward schemes, this result supports the conjecture that, within the range where GreedyMini is feasible to run, it generates minimizers with the density close to the lower bound. Based on this conjecture, we present in Fig. 5 the landscape of density factors of our best binary minimizers as an approximation of such a landscape of minimum densities. The main features of this landscape are flat “highlands” (w≥k−1), gradually ascending as *w* grows over *k*, bumpy “lowlands” (w≤k−3), and a steep step between them (k−3≤w≤k−1).

## 5 Discussion

In this paper, we tackled the problem of generating a minimizer that achieves low expected or particular density. We presented GreedyMini, a toolkit of novel methods to generate low-density minimizers, to improve a given minimizer, to extend to larger alphabet, *k*, and *w*, and to measure the density of a given minimizer. With these methods, we devised pipelines to generate low-density DNA minimizers for given (k,w) combinations, with the key role played by two variants of a greedy algorithm (GreedyE and GreedyP for expected and particular density, respectively).

We evaluated the performance of the minimizers generated by GreedyMini pipelines by calculating the achieved expected and particular densities, and conclude that GreedyMini pipelines generate minimizers that achieve the lowest density compared to all existing minimizer methods over a wide range of (k,w) combinations. This range includes the parameters used by popular HTS tools, such as Minimap2 (Li 2018), Kraken 2 (Wood *et al.* 2019), KMC 3 (Kokot *et al.* 2017), and several others. Moreover, we observed that the achieved densities are very close to the theoretical lower bound in that range, and even hit the lower bound for forward schemes for the binary alphabet and several (k,w) pairs.

The main limitation of GreedyMini is the exponential dependence on w+k of the time needed to build a minimizer by GreedyE. Thanks to our novel minimizer-to-minimizer transformation to larger alphabets (Extend-σ), this dependence is only $O(2^{2k}+w2^{w+k})$ per run of GreedyE. This is an acceptable construction time for a wide range of practical values of *k* and *w*. This range is further extended to larger *k* values due to Extend-k and to larger *w* values by GM-improve and trivial *w*-extensions. The use of Extend-σ reduces the exponential dependence of the space complexity in *k* to just $O(2^{k})$, which is a feasible limitation. For k=20, the lookup table occupies $4\times 2^{20}$ bytes (=4MB), which surely fits in the cache memory, where an access operation takes O(1) time under many reasonable models. Indeed, we showed that the *k*-mer rank-retrieval time for such a lookup table is comparable to that of the computationally trivial XOR hash function. For larger *k*, one can generate a minimizer for k=20 and use Extend-k multiple times, keeping the size of the lookup table within the same 4MB.

Our study raises several open questions. First, for which (σ,k,w) combinations minimizers are optimal among all forward schemes? Second, what is the complexity of the minimum-density problem, and can we generate minimum-density minimizers efficiently? Third, are there theoretical guarantees for GreedyE, e.g. whether the obtained density (assuming α=1) is *always* below average? Fourth, is there an algorithm that returns the rank of a *k*-mer in the order generated by GreedyE without storing the order explicitly?

There are several directions for future work. First, we plan to plug our new minimizers in schemes, such as strobemers (Sahlin 2021), to improve the density and study the impact on other metrics, such as conservation. Second, we plan to extend GreedyMini to complete any partially constructed order to a UHS order, such as an order defined on a double-decycling set. Third, we plan to test *k*-mer data structures to store only the *k*-mer ranks of the UHS instead of our array implementation, to see if they can save space without increasing *k*-mer rank-retrieval time. Fourth, we plan to incorporate our new minimizers in existing HTS algorithms and data structures and study the impact on their performance in terms of runtime and memory usage.

## Author contributions

Conceptualization: S.G., A.S. Formal analysis, Methodology: S.G., M.K., A.S. Project administration: Y.O., A.S. Software: I.T., A.S. Supervision: Y.O. Validation: I.T., A.S. Visualization: I.T. Writing—original draft: I.T., Y.O., A.S. Writing—review & editing: all authors

## Supplementary Material

btaf251_Supplementary_Data

## Data Availability

The data underlying this article are available in the article and in its online supplementary material.
