# Supplementary material for: GreedyMini: generating low-density DNA minimizers
Source: Bioinformatics. 2025 Jul 15;41(Suppl 1):i275–84. doi: 10.1093/bioinformatics/btaf251 (PMC12261476; doi:10.1093/bioinformatics/btaf251)
Supplement: btaf251_Supplementary_Data [file btaf251_supplementary_data.zip › btaf251_Supplementary_Data/Shur.4.sup.1.pdf]

Supplementary Notes

S1. Preliminaries

S1.1. Strings as integers

In our algorithms, we view k-mers and explicitly stored windows as **integers written in σ-ary** (padded with leading zeroes to the required length). As a result, many string operations, like extracting a k-mer in a given position, become unit-cost integer operations. In Algorithms S7 and S8, the integer operations are made explicit; the shift operation $v \gg_\sigma n$ deletes the last n digits (characters) of v.

S1.2. Tries and reversed tries

Given a set U of strings, its *trie* (or *prefix tree*) $\mathcal{T}_U$ is defined as follows. The set of nodes is the set of all prefixes of the strings in U; the set of edges is the set of all ordered pairs (z, za) of nodes, where $a \in \Sigma$. Similarly, the *reversed trie* $\mathcal{T}_U^{\leftarrow}$ has all *suffixes* of the strings in U as nodes and all ordered pairs of nodes of the form (z, az) as edges.

We use tries and reversed tries of sets of $(k+w)$-windows, computing node characteristics during a depth-first search (DFS) without explicitly storing the whole tree. We assume that each node z of a trie (respectively, reversed trie) with $|z| \geq k$ is labeled by its k-suffix (respectively, k-prefix).

S1.3. Relation between proofs of theorems

The algorithms proving Theorems 1, 2, and 5 share common ideas. We present these ideas from the simplest to the hardest case, so we first prove Theorem 5, then Theorem 2, and finally Theorem 1. After that, we prove Theorems 3 and 4, which also share common ideas in the proofs.

S2. Proof of Theorem 5

To calculate the density $d_{(\rho,w)}$, we count the $(w+k)$-windows charged by ρ and divide the result by σ^{w+k}. We focus on counting charged windows, doing a separate count for windows that are *prefix-charged* (the minimal k-mer is a prefix) and *suffix-charged* (the minimal k-mer is a unique suffix). Two different approaches, described below, result in the algorithms DenDFS and DenDP, reaching the complexity stated in Theorem 5 (a) and (b), respectively.

S2.1. Algorithm DenDFS

Let U be the set of all windows with some k-prefix u. The trie $\mathcal{T}_U$ of U consists of the path from the root to u and a complete σ-ary tree of depth w, rooted at u. In order to count prefix-charged windows with the prefix u, we run a recursive DFS on this complete subtree by calling $\mathsf{DFSp}(u, u, 0, \mathsf{rank}_\rho(u))$ (Algorithm S1).

With each node z we associate the minimum rank r_z of a k-mer occurring in z, i.e., $r_z = \min\{\mathsf{rank}_\rho(u') \mid u' \text{ occurs in } z\}$. If z is internal, it recursively calls all its children; the minimum rank for a child za is computed as $\min\{r_z, \mathsf{rank}_\rho(z_a)\}$, where z_a is the k-suffix of za. After that, z returns the number of prefix-charged windows in its subtree, summing up the values returned by its children. If z is a leaf, i.e., a window in U, it returns 1 if it is prefix-charged (which means exactly $r_z = \mathsf{rank}_\rho(u)$) and 0 otherwise.

Algorithm S1 : function DFSp for recursive count of prefix-charged $(w+k)$-windows

Require: k-prefix u, current node z, depth d, minimum rank r; global $\sigma, k, w, \mathsf{rank}_\rho$
Ensure: number of prefix-charged windows with the prefix z for the minimizer (ρ, w)

if $d < w$ **then** ▷ z is internal
  $c \leftarrow 0$ ▷ counter of charged windows
  for $a \in \Sigma$ **do** ▷ call children
    $z_a \leftarrow$ k-suffix of za
    $c \leftarrow c + \mathsf{DFSp}(u, za, d+1, \min\{r, \mathsf{rank}_\rho(z_a)\})$
  end for
  return c
else ▷ z is a leaf
  return $[r = \mathsf{rank}_\rho(u)]$
end if

The call $\mathsf{DFSp}(u, u, 0, \mathsf{rank}_\rho(u))$ spends $O(\sigma)$ time per internal node of $\mathcal{T}_U$ and $O(1)$ time per leaf, which is $O(\sigma^w)$ in total. It stores $O(1)$ numbers per active recursive call, which means $O(w)$ space in total.

We count suffix-charged windows with the suffix u in a symmetric way, using a recursive function DFSs on the reversed trie $\mathcal{T}_{U'}^{\leftarrow}$, where U' consists of all $(w+k)$-windows with the suffix u. The difference with DFSp is that a leaf z returns 1 if z contains, apart from the suffix u, no k-mers of rank smaller than or **equal to** $\mathsf{rank}_\rho(u)$. To ensure correct processing, we set the value returned by a leaf z to $[r_z = \mathsf{rank}_\rho(u) + 1]$ and start the search by calling $\mathsf{DFSs}(u, u, 0, \mathsf{rank}_\rho(u) + 1)$. This call obviously satisfies the complexity bounds computed above for DFSp.

Algorithm DenDFS calls $\mathsf{DFSp}(u, u, 0, \mathsf{rank}_\rho(u))$ and $\mathsf{DFSs}(u, u, 0, \mathsf{rank}_\rho(u) + 1)$ for each ranked k-mer u and sums up the results to get the number of windows charged by ρ. The algorithm spends $O(|H|\sigma^w)$ time and $O(w)$ space, as required by statement (a).

S2.2. Algorithm DenDP

Recall that *order-k deBrujin graph over* Σ is a directed σ-regular graph, having all σ-ary k-mers as nodes and all pairs (au, ub), where $u \in \Sigma^{k-1}$, $a, b \in \Sigma$, as edges. If an edge (au, ub) is labeled by b, the graph becomes a deterministic finite automaton $\mathcal{B}$. We view $\mathcal{B}$ as a *transition table* with rows indexed by k-mers and columns indexed by letters; the entry $\mathcal{B}[u, a]$ contains the successor of u by the letter a.

We complete the UHS order ρ to a linear order $\bar{\rho}$, assigning the remaining ranks lexicographically. Then we replace all elements and all row indices in $\mathcal{B}$ with their $\bar{\rho}$-ranks, and sort the rows. The resulting table is referred to as $\mathcal{B}_\rho$.

To count $(w+k)$-windows charged due to some k-mer u, we proceed by dynamic programming. Let pref_r be a two-dimensional table such that $\mathsf{pref}_r[i, j]$ is the number of strings of length $k+j$ having the k-prefix of rank r, the k-suffix of rank i, and all other k-mers of rank at least r. Then the number of $(w+k)$-windows prefix-charged due to rank-r k-mer is $\sum_{i \geq r} \mathsf{pref}_r[i, w]$. Similarly, let suff_r be a two-dimensional table such that $\mathsf{suff}_r[i, j]$ is the number of strings of length $k+j$ having the k-suffix of rank i and all other k-mers of rank greater than r. Then the number of $(w+k)$-windows suffix-charged due to rank-r k-mer is $\mathsf{suff}_r[r, w]$.

Note that $\mathsf{pref}_r[i, 0] = [i = r]$, $\mathsf{suff}_r[i, 0] = 1$, and the DP rules for pref_r and suff_r are almost the same. Namely, $\mathsf{pref}_r[i, j+1] = \sum_\ell \mathsf{pref}_r[\ell, j]$, where the summation is over all ranks $\ell \geq r$ such that the k-mer of rank i is a successor of the k-mer of rank ℓ. The rule is computed by the function DPp (Algorithm S2), which uses the transition table $\mathcal{B}_\rho$ to propagate the counts from the j'th column to the $(j+1)$th column along the edges of the deBrujin graph. The DP rule for suffixes looks the same: $\mathsf{suff}_r[i, j+1] = \sum_\ell \mathsf{suff}_r[\ell, j]$; the only difference is that the inequality for ℓ is strict: $\ell > r$. This rule is computed by the function DPs (Algorithm S3).

Algorithm S2 : function DPp for count of prefix-charged $(w+k)$-windows with a prefix of a given rank

Require: rank r; global $\sigma, k, w, \mathcal{B}_\rho$
Ensure: number of prefix-charged windows with the prefix of rank r for the minimizer (ρ, w)

$\mathsf{pref}[0..\sigma^k - 1, 0..w] \leftarrow$ zero matrix
$\mathsf{pref}[r, 0] \leftarrow 1$
for $j \leftarrow 1, \ldots, w$ **do**
  for $i \leftarrow r, \ldots, \sigma^k - 1$ **do**
    for $a \in \Sigma$ **do** ▷ propagate the current count of i to its successors
      $\mathsf{pref}[\mathcal{B}_\rho[i, a], j] \leftarrow \mathsf{pref}[\mathcal{B}_\rho[i, a], j] + \mathsf{pref}[i, j-1]$
    end for
  end for
end for
$c \leftarrow 0$
for $i \leftarrow r, \ldots, \sigma^k - 1$ **do**
  $c \leftarrow c + \mathsf{pref}[i, w]$ ▷ summing prefix-charged windows over all suffixes
end for
return c

Algorithm S3 : function DPs for count of suffix-charged $(w+k)$-windows with a suffix of a given rank

Require: rank r; global $\sigma, k, w, \mathcal{B}_\rho$
Ensure: number of suffix-charged windows with the suffix of rank r for the minimizer (ρ, w)

$\mathsf{suff}[0..\sigma^k - 1, 1..w] \leftarrow$ zero matrix
$\mathsf{suff}[0..\sigma^k - 1, 0] \leftarrow$ column of 1's
for $j \leftarrow 1, \ldots, w$ **do**
  for $i \leftarrow r+1, \ldots, \sigma^k - 1$ **do**
    for $a \in \Sigma$ **do** ▷ propagate the current count of i to its successors
      $\mathsf{suff}[\mathcal{B}_\rho[i, a], j] \leftarrow \mathsf{suff}[\mathcal{B}_\rho[i, a], j] + \mathsf{suff}[i, j-1]$
    end for
  end for
end for
return $\mathsf{suff}[r, w]$ ▷ all suffix-charged windows have the same suffix (of rank r)

Algorithm S2 works in $O(w\sigma^k)$ time (recall that we assume σ to be a constant) and uses $O(\sigma^k)$ space, as just two columns of the DP table are stored at every moment. The same bounds apply for counting suffix-charged windows.

The algorithm DenDP creates the table $\mathcal{B}_\rho$ and then calls DPp(r) and DPs(r) for each rank r assigned in ρ. As $\mathcal{B}_\rho$ can be trivially computed from rank_ρ in $O(\sigma^k)$ time and space, DenDP works in $O(|H|w\sigma^k)$ time and $O(\sigma^k)$ space, as required in statement (b). Theorem 5 is proved.

Remark S1 *An additional feature of the dynamic programming algorithm* DenDP *is that while computing $d_{(\rho,w)}$ it can, within the same time and space bounds, report the densities of* ***all*** *minimizers (ρ, w') with $w' \leq w$.*

S3. Proof of Theorem 2

Let ρ be a UHS order for w, H be its UHS, and r be a rank such that both r and $r{+}1$ are assigned in ρ. Let ρ' be the order obtained from ρ by swapping k-mers u and u' such that $\mathsf{rank}_\rho[u] = r$, $\mathsf{rank}_\rho[u'] = r+1$. We define the *cost function* $\mathsf{cost}(\rho, w, r) = \sigma^{k+w}(d_{(\rho',w)} - d_{(\rho,w)})$, equal to the difference between the numbers of $(w+k)$-windows charged by ρ' and by ρ. We focus on the efficient computation of $\mathsf{cost}(\rho, w, r)$, since all other operations in the function Swap are straightforward (Algorithm S4). Similar to Theorem 5, we present two algorithms computing the cost function: the DFS-based algorithm and the DP-based algorithm are used to prove the statements (a) and (b) of Theorem 2, respectively. Both algorithms analyse the difference between ρ and ρ', avoiding direct computation of $d_{(\rho',w)}$.

Algorithm S4 : function Swap for swapping two k-mers with consecutive ranks

---

Require: array $\mathsf{rank} = \mathsf{rank}_\rho$ for a UHS order ρ for w, rank r; global σ, k, w
Ensure: array rank after an attempted swap of ranks r and $r{+}1$

$u \leftarrow \rho(r)$; $u' \leftarrow \rho(r{+}1)$
$c \leftarrow \mathsf{cost}(\rho, w, r)$
if $c < 0$ **then**
    swap the elements $\mathsf{rank}[u]$ and $\mathsf{rank}[u']$
else if $c = 0$ **then**
    swap the elements $\mathsf{rank}[u]$ and $\mathsf{rank}[u']$ with probability 0.5
end if
return rank

S3.1. Algorithm SwapDFS

In this section, compute $\mathsf{cost}(\rho, w, r)$ by considering only those $(w{+}k)$-windows that could have different status (free/charged) for ρ and ρ'. If a window v changed its status, then its minimum-rank k-mer in ρ is u and its minimum-rank k-mer in ρ' is u'. Hence v contains both u and u'; moreover, either u or u' is a prefix and/or a suffix of v. Therefore, it is sufficient to check four sets of windows: those with prefix u, prefix u', suffix u, and suffix u'. The number of windows in these sets is $O(\sigma^w)$.

Let U be the set of all windows with the prefix u and let $v \in U$. If v contains a k-mer of rank $< r$, then it has the same status for both ρ and ρ'. Otherwise, it is charged by ρ; this charged window is free for ρ' if it contains u', but not as a unique suffix. Respectively, it suffices to count the windows that are charged by ρ and free for ρ', as the opposite change of status is impossible. Similar to the proof of Theorem 5, we make use of the trie $\mathcal{T}_U$ of U. It consists of a path from the root to u, followed by a complete σ-ary tree of depth w. We additionally assign to each internal node z a flag indicating whether u' occurs in z and run a DFS on the complete subtree, calling the function $\mathsf{pr\_cost}(\mathsf{rank}_\rho, r, u, u', u, 0, \mathsf{False})$ (Algorithm S5).

During the DFS, each node returns the number of $(w+k)$-windows in its subtree that are charged by ρ and free for ρ'. For a node z with the label z', if $\mathsf{rank}[z'] < r$, we return 0. Otherwise, the processing depends on the type of the node. Let z be internal. If $z' = u'$, we set the flag; otherwise, we copy its value from the parent of z. After that, we recursively call all children of z and return the sum of the values they returned. Now let z be a leaf. It returns 1 if the flag of its parent is set and 0 otherwise. Clearly, the root u of the subtree returns the required value. The processing time is $O(\sigma^w)$ and the space is $O(w)$, as we store only $O(1)$ numbers per each of $O(w)$ active recursive calls. We then process the set U' of windows with the prefix u' in the same way as U, calling $\mathsf{pr\_cost}(\mathsf{rank}_\rho, r, u', u, u', 0, \mathsf{False})$ to count the windows that are charged by ρ' and free for ρ.

Algorithm S5 : function pr_cost for recursive count of status-changing k-mers

---

Require: array rank, rank r, k-mers u, u' to swap, current node with k-suffix z and depth d, flag f (set if u' is already seen); global σ, k, w
Ensure: number c of windows with prefix u, in the subtree of the current node, that become free if u and u' swapped

if $\mathsf{rank}[z] < r$ **then** ▷ status of windows containing z is independent of u, u'
    return 0
end if
if $d = w$ **then** ▷ current node is a leaf (window)
    return $[f]$
else ▷ current node is internal
    $f \leftarrow f \vee (z = u')$
    $c \leftarrow 0$
    for $a \in \Sigma$ **do** ▷ recursive call for children
        $c \leftarrow c + \mathsf{pr\_cost}(\mathsf{rank}, r, u, u', (\sigma \cdot z) \bmod \sigma^k + a, d+1, f)$
    end for
end if
return c

Consider the set $\bar{U}$ of the windows with the suffix u. The options for $v \in \bar{U}$ are the following. If v contains a k-mer of rank $< r$ or it does not contain u', then it has the same status in ρ and ρ'. If v has the prefix u or u', we ignore it to avoid double count. Otherwise, v is free for ρ' and charged by ρ if and only if it has a single occurrence of u. We process $\bar{U}$ similar to U, counting the windows that are charged by ρ and free for ρ', but in the **reversed** trie $\mathcal{T}^{\leftarrow}_{\bar{U}}$. We use a DFS over the complete subtree of $\mathcal{T}^{\leftarrow}_{\bar{U}}$ rooted at u and adjust the processing of a node according to the conditions listed above, getting the function $\mathsf{sf\_cost}$ (Algorithm S6). It has the same space and time bounds as $\mathsf{pr\_cost}$, and the set $\bar{U}'$ of the windows with the suffix u' is processed in exactly the same way as $\bar{U}$. Finally, $\mathsf{cost}(\rho, w, r) = \mathsf{pr\_cost}(\mathsf{rank}_\rho, r, u', u, u', 0, \mathsf{False}) + \mathsf{sf\_cost}(\mathsf{rank}_\rho, r, u', u, u', 0, \mathsf{False}) - \mathsf{pr\_cost}(\mathsf{rank}_\rho, r, u, u', u, 0, \mathsf{False}) - \mathsf{sf\_cost}(\mathsf{rank}_\rho, r, u, u', u, 0, \mathsf{False})$ is computed in $O(\sigma^w)$ time and $O(w)$ space. Adding $O(\sigma^k)$ time for finding the k-mers u and u' (Algorithm S4, line 1), we get the complexity bounds of Theorem 2(a).

Algorithm S6 : function $\mathsf{sf\_cost}$ for recursive count of status-changing k-mers

Require: array rank, rank r, k-mers u, u' to swap, current node with k-prefix z and depth d, flag f (set if u' is already seen); global σ, k, w

Ensure: number c of windows with unique suffix u, in the subtree of the current node, that become free if u and u' swapped

if $(\mathsf{rank}[z] < i) \vee ((d > 0) \wedge (z = u))$ **then** ▷ status of windows is independent of u, u'
    return 0
end if
if $d = w$ **then** ▷ current node is a leaf (window)
    return $[f \wedge (z \neq u')]$ ▷ windows with prefix u' are processed using Alg. S5 and hence ignored here
else ▷ current node is internal
    $f \leftarrow f \vee (z = u')$
    $c \leftarrow 0$
    for $a \in \Sigma$ **do** ▷ recursive call for children
        $c \leftarrow c + \mathsf{sf\_cost}(\mathsf{rank}, i, u, u', (a\sigma^k + z) \gg_\sigma 1, d+1, f)$
    end for
end if
return c

Algorithm $\mathsf{SwapDFS}$ runs multiple iterations of local search. Each iteration calls $\mathsf{Swap}(\rho, w, r)$ for the current UHS order ρ and a randomly chosen rank r; within this call, the cost function is computed by the depth-first search as described above.

S3.2. Algorithm SwapDP

From Section S2, we use the function DPp counting $(w+k)$-windows prefix-charged due to a k-mer of a given rank (Algorithm S2), and its counterpart DPs for suffix-charged windows (Algorithm S3). Let $c(\rho, w, i)$ be the number of $(w+k)$-windows charged by ρ due to the rank-i k-mer. Then $\mathsf{cost}(\rho, w, r) = c(\rho', w, r) + c(\rho', w, r+1) - c(\rho, w, r) - c(\rho, w, r+1)$. Accordingly, we compute $c(\rho, w, r) = \mathsf{DPp}(r) + \mathsf{DPs}(r)$ and $c(\rho, w, r+1) = \mathsf{DPp}(r+1) + \mathsf{DPs}(r+1)$ using the transition table $\mathcal{B}_\rho$, then edit $\mathcal{B}_\rho$ to get $\mathcal{B}_{\rho'}$, and compute $c(\rho', w, r)$, $c(\rho', w, r+1)$ in the same way, but with the table $\mathcal{B}_{\rho'}$. Note that given $\mathcal{B}_\rho$, ρ, and rank_ρ, the transition table $\mathcal{B}_{\rho'}$ can be computed in constant time. Indeed, we take $u = \rho(r)$, compute all its predecessors by $O(1)$ arithmetic operations, extract their ranks from rank_ρ and replace r with $r+1$ in the rows of $\mathcal{B}_\rho$ corresponding to these ranks. Then we do the same with $u' = \rho(r+1)$, and replace $r+1$ with r in the obtained rows. Finally we swap the rows r and $r+1$, getting $\mathcal{B}_{\rho'}$.

In this way, $\mathsf{cost}(\rho, w, r)$ is computed within the complexity bounds of Algorithm S2, i.e., in $O(w\sigma^k)$ time and $O(\sigma^k)$ space. Then we immediately have statement (b) of Theorem 2.

Algorithm SwapDP runs multiple iterations of local search. Before the first iteration, it spends $O(\sigma^k)$ time to compute $\mathcal{B}_{\rho_0}$ for the input UHS order ρ_0. Each iteration calls $\mathsf{Swap}(\rho, w, r)$ for the current UHS order ρ and a randomly chosen rank r; within this call, the cost function is computed by the dynamic programming algorithms DPp and DPs as described above. The changes in the transition table made during this computation are retained if the swap was successful and reversed otherwise.

S4. Proof of Theorem 1

As with Theorems 2 and 5, both DFS-based and DP-based solutions are possible. For an unranked k-mer u, counting $\mathsf{score}(u) = |Y_u|/|X_u|$ can be done in $O(w\sigma^k)$ time by variants of the functions DPp (Algorithm S2) and DPs (Algorithm S3). However, this means $O(w\sigma^{2k})$ time per iteration, which is rather inefficient. Because of this, we stick to the DFS solution.

We maintain arrays X, Y, and rank, each of size σ^k. For every k-mer u, the counters $\mathsf{X}[u]$ and $\mathsf{Y}[u]$ store the current values of $|X_u|$ and $|Y_u|$, respectively; rank is used to store the assigned ranks over all k-mers. To initialize X and Y it suffices to loop through all windows, incrementing the counters of all k-mers occurring in the current window. This can be done in $O(w\sigma^{w+k})$ time in a trivial way. Then at most σ^k iterations follow, each consisting of two steps: choosing the k-mer to assign rank to and updating the arrays X, Y (for convenience, the rank is assigned *after* the updates). Computing the pool of low-scored k-mers and choosing a random k-mer from it can be done in time proportional to the size of X and Y, i.e., $O(\sigma^k)$.

Assume that the k-mer u is chosen and consider the update step (Algorithm S7). For every $i \in [0, w{+}1)$, exactly σ^w windows contain u at position i. Hence, the number of windows containing u is $O(w\sigma^w)$, and to fit into the time bound we need to spend amortized $O(1)$ time per such window while skipping all windows that do not contain u. We group windows containing u in *u-blocks*. A u-block is a set of all windows v sharing the same prefix pu, where p is followed by the leftmost u in v. The number of u-blocks

with the leftmost u starting at position i equals the number of options for p, which is at most σ^i. Hence, the total number of u-blocks is at most $\sum_{i=0}^{w} \sigma^i = O(\sigma^w)$.

Algorithm S7 : function update_counts

```
Require: Arrays X, Y, rank, k-mer u; global σ, k, w
Ensure: Arrays X′, Y′ updated from X and Y
v, d ← nextblock(0, u)                                   ▷ compute the first u-block (Algorithm S8)
while v < ∞ do                                           ▷ current block exists
    seen ← ∅
    for i ← 0 to d − 1 do                                ▷ processing k-mers starting to the left of u
        z ← (v ≫_σ (w−i)) mod σ^k                        ▷ k-mer starting at position i
        if rank[z] < ∞ then                              ▷ no live windows in this block, move to next
            v, d ← nextblock(((v ≫_σ (w−i)) + 1)) · σ^(w−i), u); break   ▷ compute the next u-block (Algorithm S8)
        end if
        seen ← seen ∪ {z}
    end for
    L, X′, Y′ ← DFS_count_and_update(u, d, seen, X, Y)    ▷ Algorithm S9
    for z ∈ seen do                                      ▷ subtract the number of live windows in the current block
        X′[z] ← X′[z] − L
    end for
    Y′[v ≫_σ w] ← Y′[v ≫_σ w] − L                        ▷ for the prefix of v live windows are charged
    v, d ← nextblock((v ≫_σ (w−d)) + 1)) · σ^(w−i), u)    ▷ move to the next block
end while
return X′, Y′
```

We process one block at a time, navigating between blocks with the help of an auxiliary function nextblock(v, u) (Algorithm S8): it returns the smallest window v' that is greater than the window v and contains u, or null if no such window exists. In other words, nextblock(v, u) returns the first window of the next u-block. To implement nextblock(v, u), we compute, for every $i \in [0, w{+}1)$, the smallest window v_i (if any) that is greater than v and contains u at position i. Computing v_i from v, u, i requires a constant number of arithmetic operations. Hence, nextblock(v, u) can be computed in $O(w)$ time.

Algorithm S8 : function nextblock

```
Require: window v, k-mer u; global σ, k, w
Ensure: smallest window v′ ≥ v that contains u, leftmost position d of u in v′
v′ ← ∞; d ← ∞                                            ▷ no window found yet
for i ← 0 to w do
    z ← (v mod σ^(w+k−i)) ≫_σ (w − i)                    ▷ the k-mer starting at position i in m
    if z < u then                                        ▷ replace u with z and the rest with 0's
        x ← ((v ≫_σ (w−i)) + u − z) · σ^(w−i)
    else if z > u then                                   ▷ same + add 1 to the digit preceding u
        x ← ((v ≫_σ (w−i)) + σ^k + u − z) · σ^(w−i)
    else
        return v, i                                      ▷ v contains u, the leftmost occurrence is at i
    end if
    if x < min{v′, σ^(w+k)} then                         ▷ smaller window found
        v′ ← x; d ← i
    end if
end for
return v′, d
```

Consider processing a u-block U (one iteration of the **while** cycle in Algorithm S7). The goal is to count, for each unranked k-mer u' occurring in at least one window of U, (i) the number of live windows from U containing u' and (ii) the number of live windows from U containing u' as a prefix or as a unique suffix. These numbers are then subtracted from $\mathsf{X}[u']$ and $\mathsf{Y}[u']$ respectively; in particular, $\mathsf{X}[u]$ and $\mathsf{Y}[u]$ become zeroes. Let pu be the common prefix of all windows in U, $i = |p|$, and let $\mathcal{T}_U$ be the trie of U. Each node of $\mathcal{T}_U$ is identified with a prefix of some windows from U, and leaves are identified with the windows themselves. The trie consists of a single path from the root to the node pu followed by a complete σ-ary tree of depth $w - i$ below pu. Recall that each node of this complete tree is labeled with its k-suffix.

We first check all k-mers in pu except for u; if any of them has an assigned rank, then U contains no live windows, so the processing is finished (since all numbers to be subtracted from the cells of X and Y are zeroes). Otherwise, we put all of them in

Algorithm S9 : function DFS_count_and_update

Require: k-mer z, depth d, set seen of occurred k-mers, arrays X, Y; global σ, k, w
Ensure: number L of live windows below z, arrays X', Y' updated from X and Y

```
if X[z] = 0 then                                ▷ no live windows contain z (includes the case rank(z) < ∞)
    return 0, X, Y
else
    X' ← X; Y' ← Y
    f ← z ∉ seen                                ▷ set flag f if z is a unique suffix of the current node
    if d = w then                               ▷ current node is a leaf (live window)
        if f then                               ▷ z is a unique suffix, decrement both X'[z], Y'[z]
            X'[z] ← X'[z] − 1; Y'[z] ← Y'[z] − 1
        end if
        return 1, X', Y'
    else                                        ▷ current node is internal
        if f then
            seen ← seen ∪ {z}                   ▷ add z to occurred k-mers
        end if
        ℓ ← 0                                   ▷ initialize counter of live windows
        for i ← 0 to σ − 1 do                   ▷ call DFS for children
            z' ← (σ · z) mod σ^k + i
            c, X', Y' ← DFS_count_and_update(z', d + 1, seen, X', Y')
            ℓ ← ℓ + c
        end for
        if f then                               ▷ subtract live windows from X'[z]
            X'[z] ← X'[z] − ℓ; seen ← seen \ {z}
        end if
        if d = 0 then                           ▷ z is a prefix, decrease Y'[z]
            Y'[z] ← Y'[z] − ℓ
        end if
        return ℓ, X', Y'
    end if
end if
```

a set seen of occurred k-mers, associate seen with the node pu of $\mathcal{T}_U$, and run a recursive DFS (Algorithm S9) on the complete subtree of $\mathcal{T}_U$.

When the DFS reaches a node z with the label z', we first check if z' has an assigned rank. If yes, there are no live windows in the subtree of z, so this subtree is skipped and z returns 0. Otherwise, we set a boolean flag if $z' \in$ seen. After that, if z is an internal node, we insert z' into seen if it was not there, and recursively call the children of z. When all children return values, we compute and return their sum s. If the boolean flag was not set, then before returning we delete z' from seen and subtract s from $\mathsf{X}[z']$ (if the flag *was set*, the subtraction will be done when processing the topmost occurrence of z' among ancestors of z). Finally, if z is a leaf, we just return 1; if the flag was not set, before returning we subtract 1 from both $\mathsf{X}[z']$ and $\mathsf{Y}[z']$, since z' is a unique suffix of a live window z.

When the DFS is finished, the root pu of the complete subtree returns the number $\hat{s}$ of live windows in U. Now it remains to subtract $\hat{s}$ from $\mathsf{X}[u']$ for each k-mer u' in pu (excluding u, which is already processed) and also from $\mathsf{Y}[u'']$ for the k-prefix u'' of pu. Thus, we reached the claimed goal.

When all u-blocks are processed, each value $\mathsf{X}[u']$ (respectively, $\mathsf{Y}[u']$) was decreased by the number of live windows that contain u and also u' (respectively, also u' as a prefix or as a unique suffix). Since the live windows containing u are exactly those losing the state of "live" after the current iteration, the arrays X and Y are updated correctly.

It remains to check the time and space complexity. Processing a u-block U requires $O(w)$ time for the k-mers in the common prefix pu and $O(1)$ time per node of the complete subtree of the prefix tree $\mathcal{T}_U$. As there are $O(\sigma^w)$ u-blocks and the size of a complete subtree is proportional to the number of leaves in it, each of two processing times sums up to $O(w\sigma^w)$ over all u-blocks. We also make $O(\sigma^w)$ calls to the nextblock function to move to the next u-block after finishing with the current one; as one call costs $O(w)$ time, we arrive at the same bound. Adding $O(\sigma^k)$ time to select the k-mer u and multiplying the result by the number of iterations, we get the claimed time bound.

As for the space complexity, we do not store the subtree in which we run the DFS: the navigation is by arithmetic operations, so at any moment we store the set seen of $O(w)$ size plus $O(1)$ numbers per each of $O(w)$ active calls. Adding the size of the arrays X, Y, and rank, we get the desired space bound. Theorem 1 is proved.

S5. Discussion on σ- and k-extensions

In this section we analyze whether the straightforward density-preserving σ- and k-extensions of a minimizer are minimizers.

S5.1. σ-extension

Let $f = (\rho, w)$ be a minimizer with the parameters (σ, k, w) and $\gamma > 1$ be an integer. Let $\bar{*}$ be any *projection* function from the alphabet $\{0, \ldots, \sigma\gamma - 1\}$ onto $\{0, \ldots, \sigma - 1\}$. The local scheme f_γ with the parameters $(\sigma\gamma, k, w)$ is defined as follows: in every window v, f_γ picks the position chosen by f in the *projection* $\bar{v}$ of v, obtained from v by applying $\bar{*}$ to each letter of v.

Lemma 2 f_γ is not a minimizer.

Proof Suppose that f_γ is a minimizer (π, w). By pigeonhole principle there must be a symbol $s \in \{0, \ldots, \sigma - 1\}$ such that for two distinct $i, j \in \{0, \ldots, \sigma\gamma - 1\}$ we have $s = \bar{i} = \bar{j}$. Consider the k-mer u having the maximal π-rank among k-mers with the projection s^k. Let v be a window with the projection $\bar{v} = s^{w+k-1}$ such that $v[0, k) = u$ and $v[1, k{+}1) = u' \neq u$. Then f_γ picks the position 0 while $\mathsf{rank}_\pi(u) > \mathsf{rank}_\pi(u')$. Hence $f \neq (\pi, w)$; the contradiction proves that f_γ is not a minimizer. □

S5.2. k-extension

Let $f = (\rho, w)$ be a minimizer with the parameters (σ, k, w) and $k' > k$ be an integer. The local scheme f' with the parameters (σ, k', w) is defined as follows: in every $(w + k' - 1)$-window v, f' picks the position chosen by f in the $(w + k - 1)$-prefix of v.

Lemma 3 If $w > k'$, then f' is not a minimizer.

Proof Suppose that $f' = (\rho', w)$ is a minimizer. Let $u \in \Sigma^k$, $x \in \Sigma^{k'-k}$ be such that $\mathsf{rank}_{\rho'}(ux) = 0$. As a minimizer, f' picks the starting position of ux in every $(w + k' - 1)$-window in which this k'-mer occurs. As $w > k' > k$, every two k-mers appear in some $(w + k' - 1)$-window; hence $\mathsf{rank}_\rho(u) = 0$.

Consider some k'-mer $uy \neq ux$. Since $w > k'$, there exists a $(w + k' - 1)$-window v with the prefix $uyux$. Since $\mathsf{rank}_\rho(u) = 0$, f' by definition picks the position 0 of v, while v contains the k'-mer ux of smaller ρ'-rank. This contradiction proves that f' is not a minimizer. □

If $w \leq k'$, the answer to the question whether f' is a minimizer depends on both w and ρ. In particular, if there exist $(w{+}k'{-}1)$-windows v_1 and v_2 such that (i) v_1 begins with the k'-mer ux and contains the k'-mer uy; (ii) v_2 begins with uy and contains ux; (iii) u has the lowest ρ-rank among all k-mers in the $(w + k - 1)$-prefixes of v_1 and v_2, then f' is not a minimizer. Indeed, similar to Lemma 3, f' picks the position 0 in both v_1 and v_2, and thus ux and uy cannot be compared.

The authors of [de Blasio et al., Practical universal k-mer sets for minimizer schemes. ACM-BCB 2019] used the following notion of extension: if ρ and ρ' are orders of k-mers and $(k + 1)$-mers respectively, and for every $u, u' \in \Sigma^k$, $a, b \in \Sigma$ the inequality $\mathsf{rank}_\rho(u) < \mathsf{rank}_\rho(u')$ implies $\mathsf{rank}_{\rho'}(ua) < \mathsf{rank}_{\rho'}(u'b)$, then ρ' is an extension of ρ. They claimed, in Theorem 3 of the cited paper, that $d_{(\rho',w)} \leq d_{(\rho,w)}$ for any extension ρ' of ρ. Their proof has a flaw: they claim that if the minimizer (ρ', w) picks position i in some $(w{+}k)$-window v due to a $(k{+}1)$-mer ua at this position, then the minimizer (ρ, w) picks the same position i in $v[0, w{+}k{-}1)$ due to the k-mer u. But v can contain ub, where $b \neq a$ and $\mathsf{rank}_{\rho'}(ub) > \mathsf{rank}_{\rho'}(ua)$, in a position $j < i$. In this case, (ρ, w) does not pick the position i in $v[0, w + k - 1)$, because ties are broken to the left.

The flawed proof does not imply automatically that Theorem 3 of [de Blasio et al.] fails, but our experiments show that the application of the particular extension defined in Section 3.3 in some cases increases density, so the theorem in the stated form is indeed incorrect.

S6. Proof of Theorem 3

We call a window $v \in (\Sigma \times \Gamma)^{w+k}$ *bad* if it is charged by $\rho \times \tau$ while v_Σ is free for ρ, and *good* if v is free for $\rho \times \tau$ while v_Σ is charged by ρ. Let B and G denote the sets of bad and good windows, respectively. The order $\rho \times \tau$ charges exactly $\gamma^{w+k}c + |B| - |G|$ windows, where c is the number of windows charged by ρ. Consequently, $d_{(\rho\times\tau,w)} = d_{(\rho,w)} + \frac{|B|-|G|}{(\sigma\gamma)^{w+k}}$. Next, let $B^{\leftarrow}$ and $G^{\leftarrow}$ be the sets of bad and good windows for the order $\rho^{\leftarrow} \times \tau$, respectively.

Claim 1 $|B| \leq |G^{\leftarrow}|$.

Proof of the Claim Let $v \in B$ and let u be the minimum-rank k-mer of v. Then, u_Σ has the minimum ρ-rank among the k-mers of v_Σ. By the definition of bad, v_Σ is not charged by ρ, so u_Σ is neither a prefix nor a unique suffix of v_Σ. Hence, u is a suffix of v, and u_Σ occurs more than once in v_Σ. Moreover, $\mathsf{rank}_\tau(u_\Gamma) < \mathsf{rank}_\tau(z_\Gamma)$ for each other k-mer z of v such that $z_\Sigma = u_\Sigma$. We define the $(w{+}k)$-window $x = \phi(v)$ as follows: $x_\Sigma = \overleftarrow{v_\Sigma}$, $x_\Gamma = v_\Gamma[i, w{+}k)v_\Gamma[0, i)$, where i is the starting position of the leftmost occurrence of u_Σ in v_Σ (Figure S1).

By the definition of mirror, the prefix $\overleftarrow{u_\Sigma}$ of x_Σ has the minimum $\rho^{\leftarrow}$-rank among the k-mers of x_Σ. Then, x_Σ is charged by $\rho^{\leftarrow}$. Let $z = v[i, i + k)$, $u' = x[0, k)$, $z' = x[w{-}i, w{-}i{+}k)$, and $y = x[w, w + k)$. Since $z_\Sigma = u_\Sigma$ by the choice of i, we have $\mathsf{rank}_\tau(u_\Gamma) < \mathsf{rank}_\tau(z_\Gamma)$. Since $u'_\Sigma = z'_\Sigma = \overleftarrow{u_\Sigma}$, $u'_\Gamma = z_\Gamma$, and $z'_\Gamma = u_\Gamma$, we get $\mathsf{rank}_{\rho^{\leftarrow}\times\sigma}(z') < \mathsf{rank}_{\rho^{\leftarrow}\times\sigma}(u')$. Note that $y_\Sigma \neq \overleftarrow{u_\Sigma}$ because u_Σ is not a prefix of v_Σ. As $\overleftarrow{u_\Sigma}$ has the minimum $\rho^{\leftarrow}$-rank among the k-mers of x_Σ, we have $\mathsf{rank}_{\rho^{\leftarrow}\times\sigma}(u') < \mathsf{rank}_{\rho^{\leftarrow}\times\sigma}(y)$. Since z' has smaller rank than both the k-prefix and the k-suffix of x, x is free for $\rho^{\leftarrow} \times \tau$. Then, $x \in G^{\leftarrow}$ by the definition of a good window. Hence, ϕ maps B to $G^{\leftarrow}$.

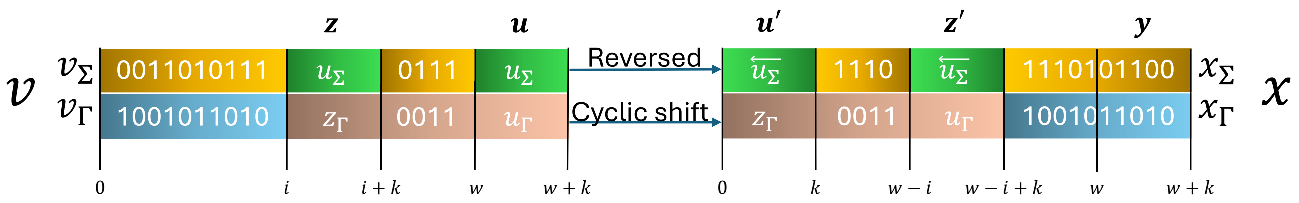

Fig. S1. Example of mapping a bad (for $\rho \times \tau$) window v to a good (for $\rho^{\leftarrow} \times \tau$) window x.

Now let $v, v' \in B$, $v \neq v'$, $x = \phi(v)$, $x' = \phi(v')$. If $v_\Sigma \neq v'_\Sigma$, then $x_\Sigma \neq x'_\Sigma$. If $v_\Sigma = v'_\Sigma$, then x_Γ, x'_Γ are obtained by the same shift of v_Γ and v'_Γ, respectively. Since $v \neq v'$, we have $v_\Gamma \neq v'_\Gamma$, and then $x_\Gamma \neq x'_\Gamma$. Therefore, in both cases $x \neq x'$. Thus, we proved that ϕ is injective. The claim now follows. □

Since $(\rho^{\leftarrow})^{\leftarrow} = \rho$, the claim implies $|B^{\leftarrow}| \leq |G|$. Then

$$d_{(\rho\times\tau,w)} + d_{(\rho^{\leftarrow}\times\tau,w)} = d_{(\rho,w)} + d_{(\rho^{\leftarrow},w)} + \frac{|B|-|G|+|B^{\leftarrow}|-|G^{\leftarrow}|}{(\sigma\gamma)^{w+k}} \leq d_{(\rho,w)} + d_{(\rho^{\leftarrow},w)}.$$

The result now follows by Lemma 1.

S7. Proof of Theorem 4

Similar to the proof of Theorem 3, we define bad and good windows. For $v \in \Sigma^{w+k}$ and $a \in \Sigma$, the window va is bad (good) for ρ_1 if it is charged (not charged) by ρ_1 while v is not charged (charged) by ρ. By B_1 and G_1 we denote the sets of bad and good windows for ρ_1, respectively. Similarly, we define bad and good windows av for ρ_2 and denote their sets by B_2 and G_2, respectively. We prove the analog of the claim from Theorem 3.

Claim 2 $|B_1| \leq |G_2|$ and $|B_2| \leq |G_1|$.

Proof of the Claim Let $va \in B_1$ and let ub be the $(k{+}1)$-mer in va of minimum ρ_1-rank (here $a, b \in \Sigma$). By the definition of ρ_1, u has the minimum ρ-rank in v. Since v is free for ρ, u is not a prefix of v. Hence, ub is a unique suffix of va; in particular, $b = a$. Then, u is a suffix of v and has other occurrences in v because v is free for ρ. Thus, v contains a $(k{+}1)$-mer ua', where $a' > a$ as $\mathsf{rank}_{\rho_1}(ua') > \mathsf{rank}_{\rho_1}(ua)$.

The window $\overleftarrow{v}$ is charged by $\rho^{\leftarrow}$ as its minimum-rank k-mer $\overleftarrow{u}$ is its prefix (but not suffix). Consider the window $a\overleftarrow{v}$. Its prefix $a\overleftarrow{u}$ has smaller ρ_2-rank than its $(k{+}1)$-suffix, as this suffix does not end with $\overleftarrow{u}$. Further, $a\overleftarrow{v}$ contains the $(k{+}1)$-mer $a'\overleftarrow{u}$ with $a' > a$ and thus $\mathsf{rank}_{\rho_2}(a'\overleftarrow{u}) < \mathsf{rank}_{\rho_2}(a\overleftarrow{u})$. Hence, $a\overleftarrow{v}$ is free for ρ_2. We thus proved $a\overleftarrow{v} \in G_2$. As the reversal of every window from B_1 belongs to G_2, we have $|B_1| \leq |G_2|$. A symmetric argument proves $|B_2| \leq |G_1|$, completing the claim. □

Let $c, c^{\leftarrow}$ be the numbers of windows charged by ρ and $\rho^{\leftarrow}$, respectively. Then, ρ_1 charges $\sigma c + |B_1| - |G_1|$ windows, and ρ_2 charges $\sigma c^{\leftarrow} + |B_2| - |G_2|$ windows. Hence, $d_{(\rho_1,w)} = d_{(\rho,w)} + \frac{|B_1|-|G_1|}{\sigma}$, $d_{(\rho_2,w)} = d_{(\rho^{\leftarrow},w)} + \frac{|B_2|-|G_2|}{\sigma}$. Substituting the inequalities from the claim, we get

$$d_{(\rho_1,w)} + d_{(\rho_2,w)} \leq d_{(\rho,w)} + d_{(\rho^{\leftarrow},w)},$$

implying the theorem in view of Lemma 1.

S8. UHS sizes of binary orders generated by GM-expected

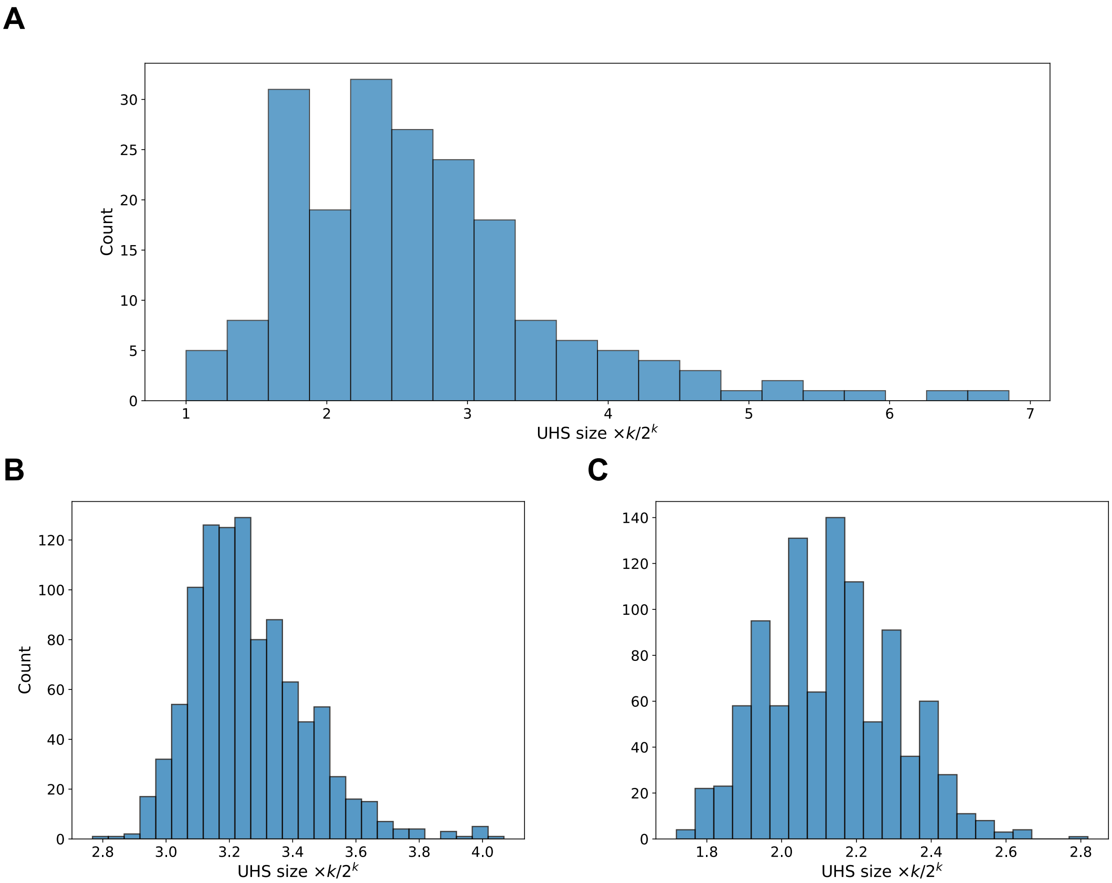

Fig. S2. Histograms of binary UHS sizes $\times k/2^k$. (A) Histogram over all binary orders generated by GM-expected in our benchmarking (Subsection 4.1). (B) Histogram over 1000 GreedyE binary orders for $(w, k) = (8, 13)$. (C) Histogram over 1000 GreedyE binary orders for $(w, k) = (13, 8)$.

S9. Runtime comparison of expected density measurement algorithms

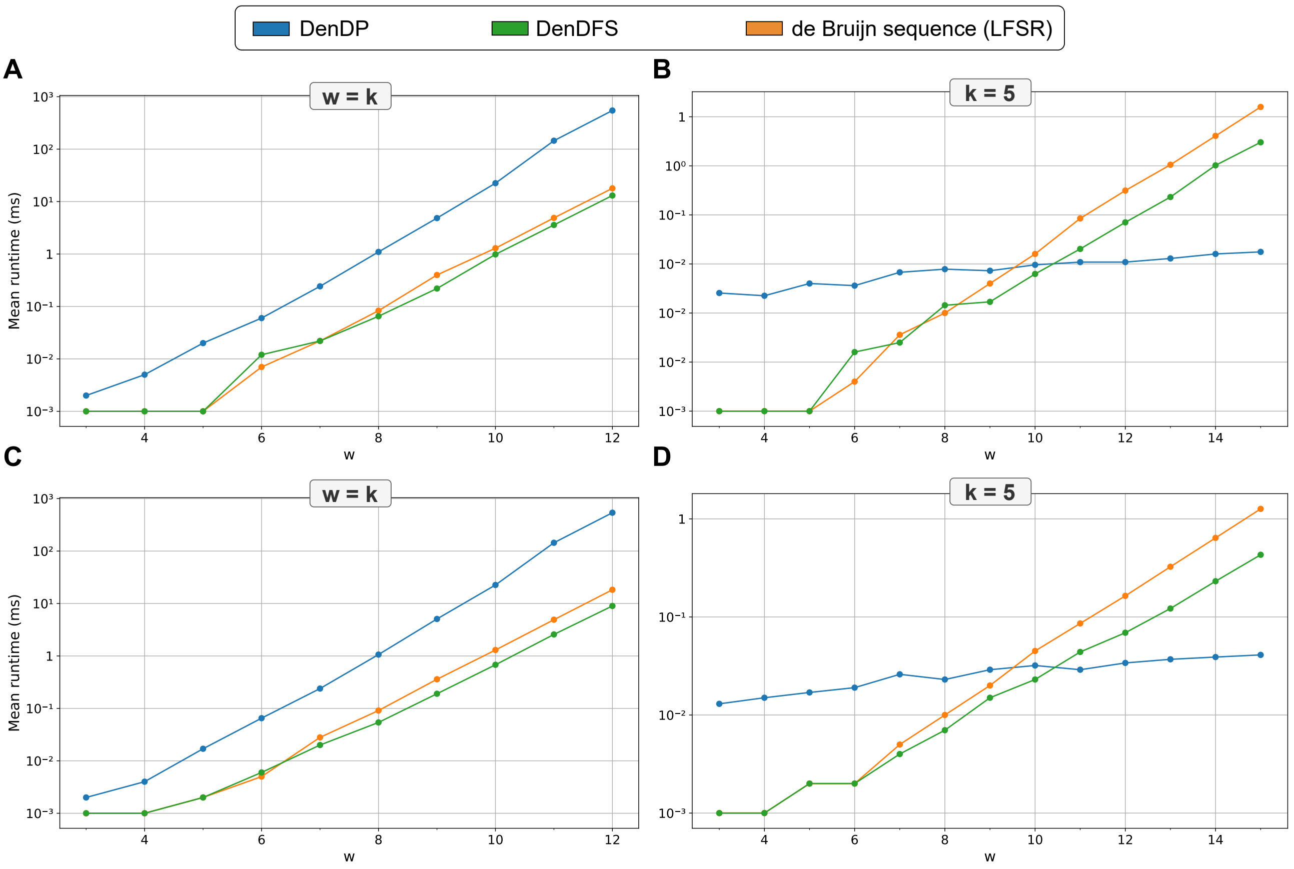

Fig. S3. Runtime of three expected density measurement algorithms averaged over 100 runs. The runtime (y-axis) is in log scale. (A-B) Measuring the expected density of a random order. (C-D) Measuring the expected density of a GM-expected order without knowing its UHS.

We compared the runtime of DenDFS and DenDP to an algorithm measuring expected density via a de Bruijn sequence. This algorithm processes all $(k+w)$-windows of a binary de Bruijn sequence of order $k+w$. After processing a window, the algorithm discards it without performing any density-related computation. Thus, the runtime of the algorithm serves as a lower bound for any density measurement based on a de Bruijn sequence. Our implementation generates consecutive windows via the linear-feedback shift register (LFSR). The time complexity of using LFSR is linear in the length of a de Bruijn sequence. For a fair comparison, we modified DenDFS and DenDP to have no knowledge of the UHS size of the input order. We compared expected density measurement of random binary orders and on GM-expected binary orders (Supplementary Table S5) for $w = k$ and for $k = 5$.

When $w = k$, DenDP has the worst theoretical complexity and naturally trails behind empirically (Supplementary Figure S3A,C), while DenDFS outperforms the de-Bruijn-sequence algorithm due to efficiently pruned DFS scans made by its subroutines DFSp and DFSs. The advantage of DenDFS is amplified when the processed order has small UHS size even though DenDFS does not know it (Supplementary Figure S3C compared to Supplementary Figure S3A). Setting $k = 5$ and running over increasing values of w, we observe the existence a point from which it's beneficial to use DenDP over the other two algorithms, owing to its time complexity being linear in w (Supplementary Figures S3B,D).

S10. GreedyMini workflows

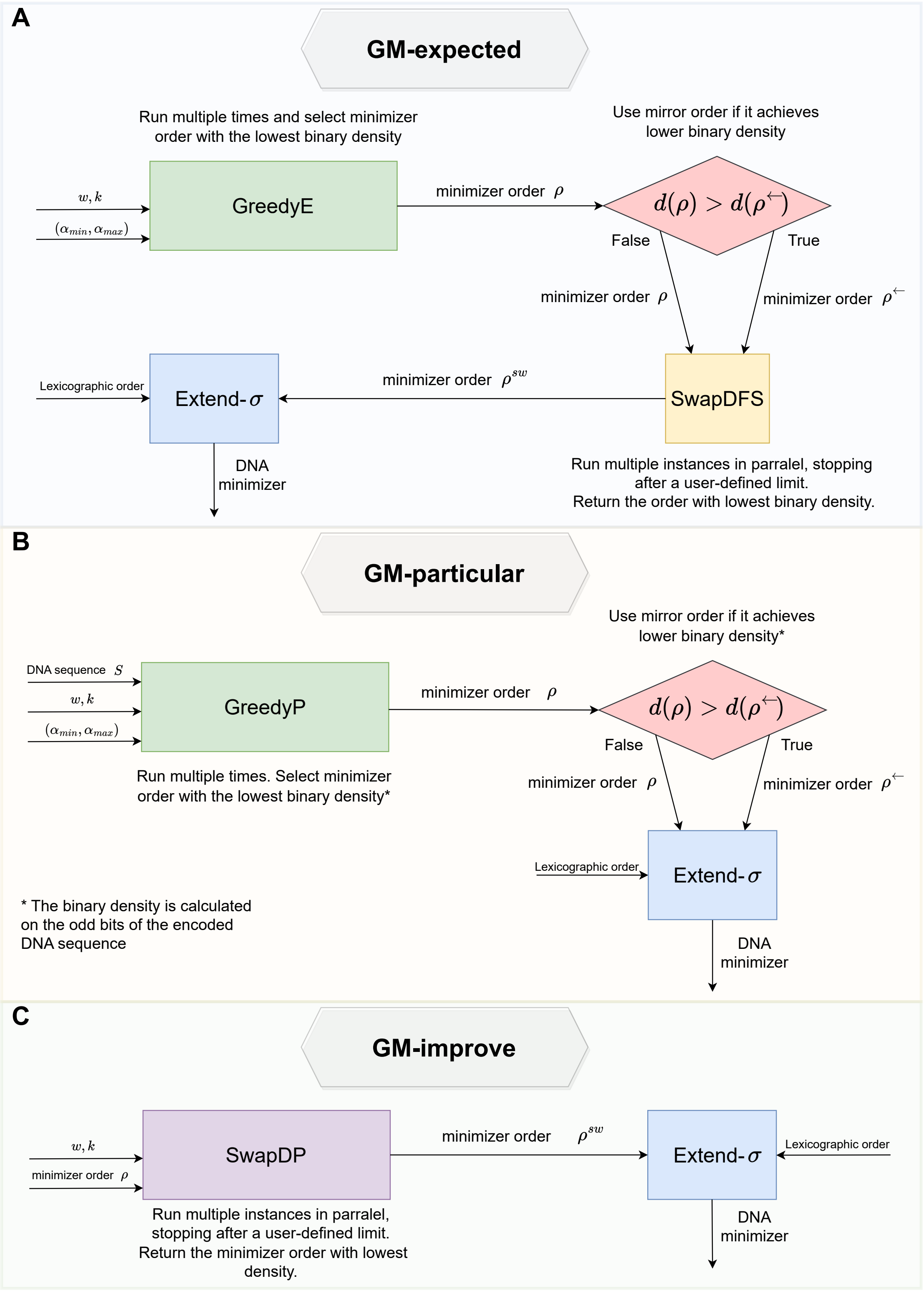

Fig. S4. Workflow illustration of (A) GM-expected pipeline to generate a low-expected-density DNA minimizer, (B) GM-particular pipeline to generate a low-particular-density DNA minimizer, and (C) GM-improve pipeline to improve a (binary) minimizer to larger w values.

S11. Running DeepMinimizer and polar sets

We failed to run DeepMinimizer [11] and polar sets [22] due to errors received when running their code. Both implementations contained unspecified file hierarchies, hard-coded paths, and lacked essential documentation, such as the required dependencies and their versions. For instances where hard-coded paths were present, we replaced them with local paths. After adjusting the paths, the following issues arose:

- **Polar sets**: We ran the following command:

  ```
  python anchor_distributed.py sequence_1M -w 12 --kmin 3
  --kmax 16 -p 10
  ```

  It raised the following error:

  ```
  Traceback (most recent call last):
    File "/home/dsi/tzionyi/polarset/anchor_distributed.py",
    line 18, in <module> assert working_dir is not None
  ```

- **DeepMinimizer**: We ran the following command:

  ```
  python run_scripts.py
  ```

  It raised the following error:

  ```
  RuntimeError: one_hot is only applicable to index tensor.
  ```

S12. Runtime statistics and user recommendations on GM-expected and GM-particular

We ran GM-expected and GM-particular on a Linux server equipped with 2 $\times$ Intel(R) Xeon(R) Gold 6338 CPUs (total cores 64) @ 2.00GHz and 512GB of RAM. We utilized 64 available cores. We report the construction runtime (Supplementary Figure S5A) and maximum memory usage (Supplementary Figure S5B) of our implementation of GM-expected over various combinations of w and k without extending k (Theorem 4).

The measured construction runtimes fit the bound of GreedyE given by Theorem 1 ($O(\sigma^{2k} + w\sigma^{k+w})$). Focusing on $w + k > 18$ to avoid effects of small values, we observe for $k > 2w$ an exponential dependence on k, while for $k \leq 2w$ we observe an exponential dependence on $w + k$. Note that we run the local search by Swap iterations with a time limit according to the runtime of GreedyMini, and hence their runtimes are equal. The maximum memory usage matches that of GreedyE and Swap by Theorems 1 and 2 ($O(\sigma^k + w)$). Note that due to the utilization of various libraries and other computational factors, our implementation of GM-expected has a baseline memory usage, and thus the increase in maximum memory usage is only observed for $k \geq 14$.

We recommend using GM-expected for $w + k \leq 32$, which took at most 1.5 days on our hardware (Supplementary Figure S5A), or for $w + k \leq 35$ if using hardware with more cores or if longer runtimes are permitted. It is also possible to run on a larger $w + k$ value by decreasing the number of GreedyE runs. While the maximum memory usage for running GM-expected is usually negligible (Supplementary Figure S5B), storing the resulting order in the cache for later use in sampling is problematic for larger values of k (Figure 4) and is dependent on hardware. We recommend using GM-expected for $k \leq 18$. For values $k > 18$ when for GM-expected it is only feasible to run with $w < k$, we recommend using GM-expected orders of smaller k' and plugging them into the mod-minimizer scheme [15]. For cases where $w + k$ is lower than the feasibility limit, we recommend increasing the number of GreedyE runs, and/or the time allowed for SwapDFS. If $w + k$ is greater than the feasibility limit, we recommend either using $k' < k$ with a trivial k-extension from the minimizer built for (k', w), or using $w' < w$ with a trivial w-extension from the minimizer built for (k, w'). Whenever $k \leq 15$ and $w \leq 30$, we recommend running GM-improve instead of a trivial w-extension.

In addition, we report the construction runtime (Supplementary Figure S6A) and maximum memory usage (Supplementary Figure S6B) of our implementation of GM-particular. Over a sequence length of 1 million nucleotides, the construction runtime increased exponentially with k for $k > 12$, and increased much slower with w. The maximum memory usage increased with w more profoundly than with k.

Due to the high dependence on the input sequence, our only guideline for using GM-particular is to run it for $k \leq 18$ (using trivial k- and/or w-extensions if necessary for larger k or w, respectively) for similar arguments as above. The number of GreedyP runs can be modified based on the available computing time.

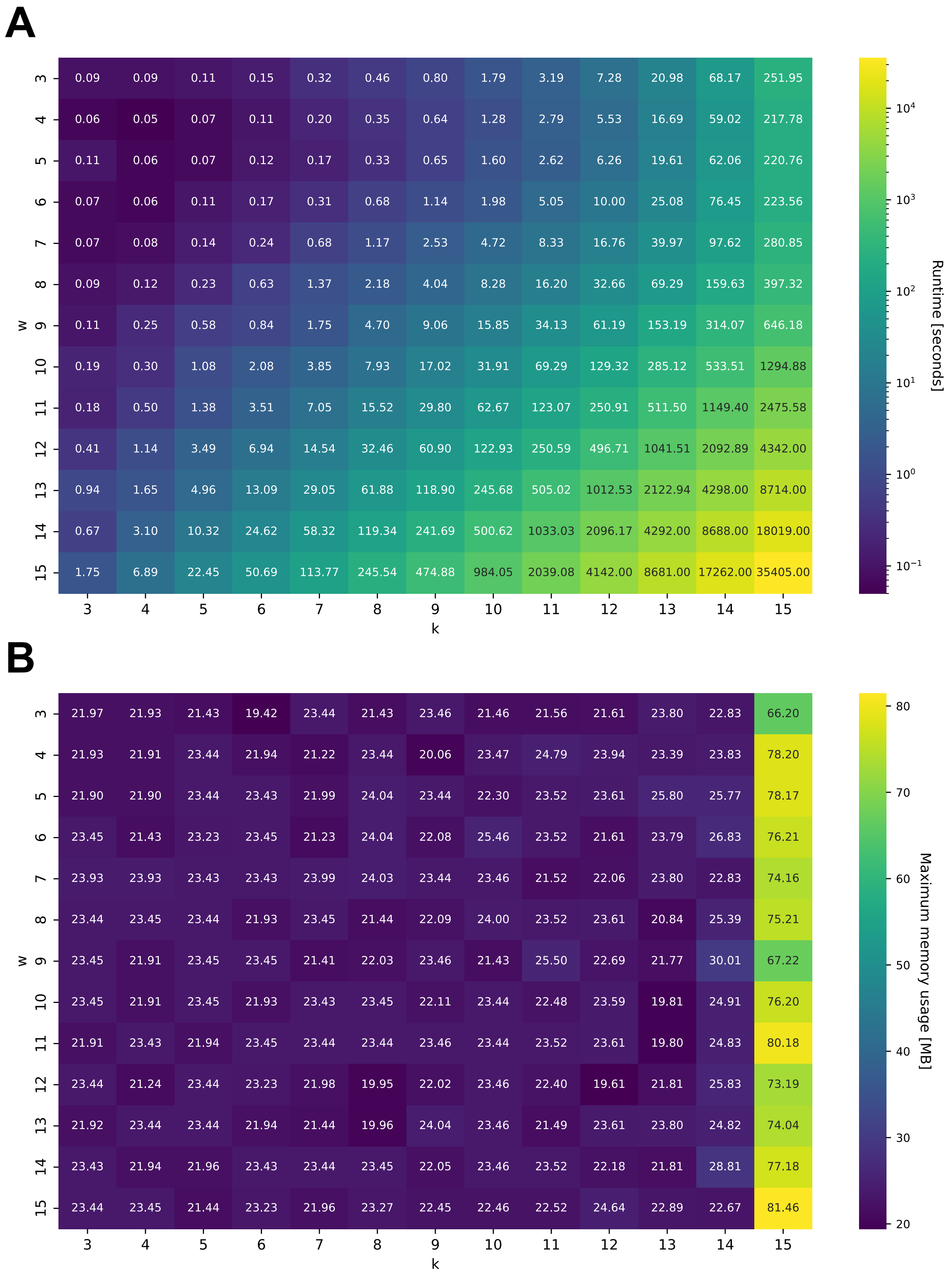

Fig. S5. Runtime (A) and maximum memory usage (B) of GM-expected on various w and k values.

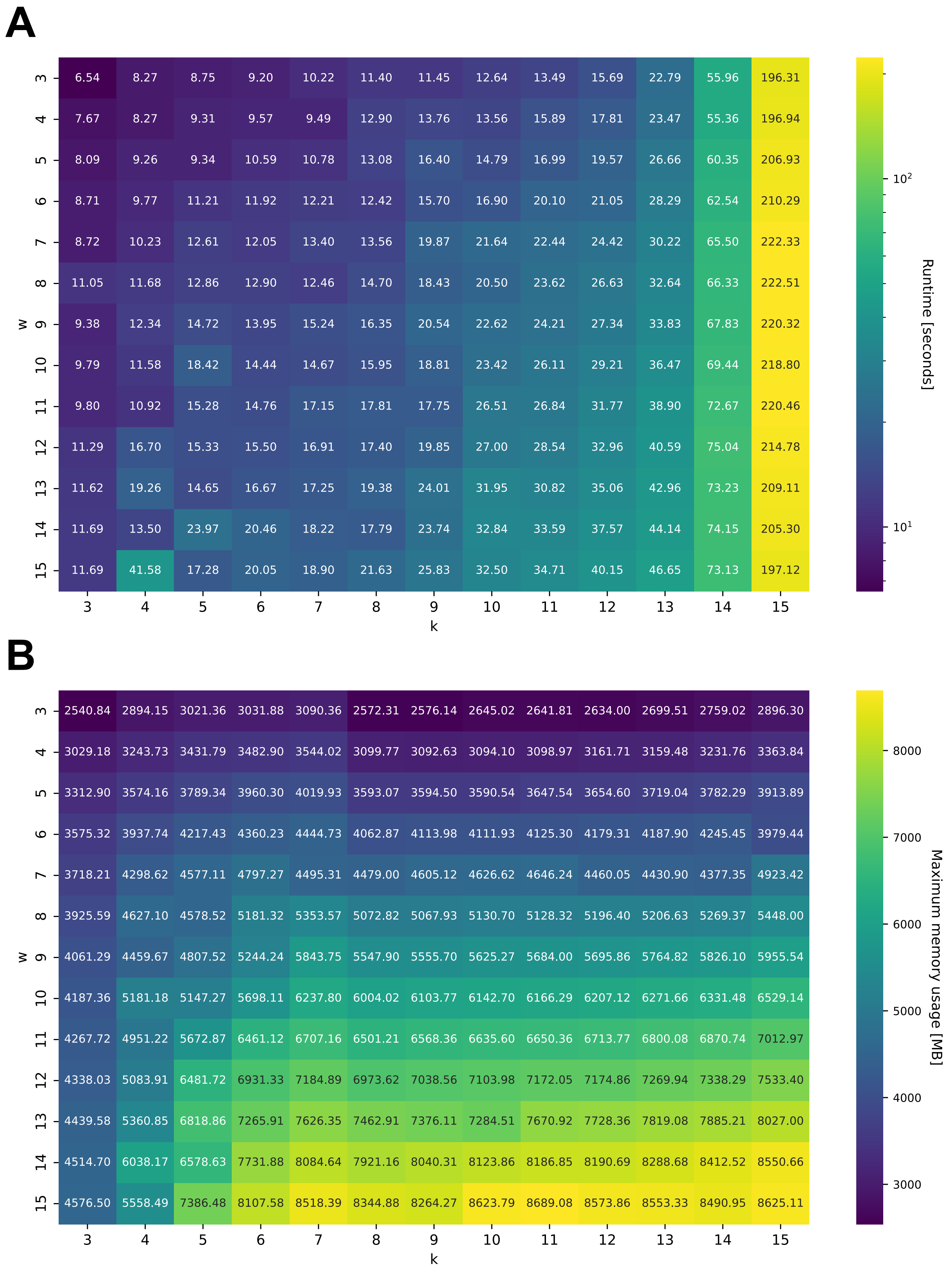

Fig. S6. Runtime (A) and maximum memory usage (B) of GM-particular on various w and k values over 1 million nucleotides from Chromosome X.

S13. Swap effectiveness on other minimizer orders

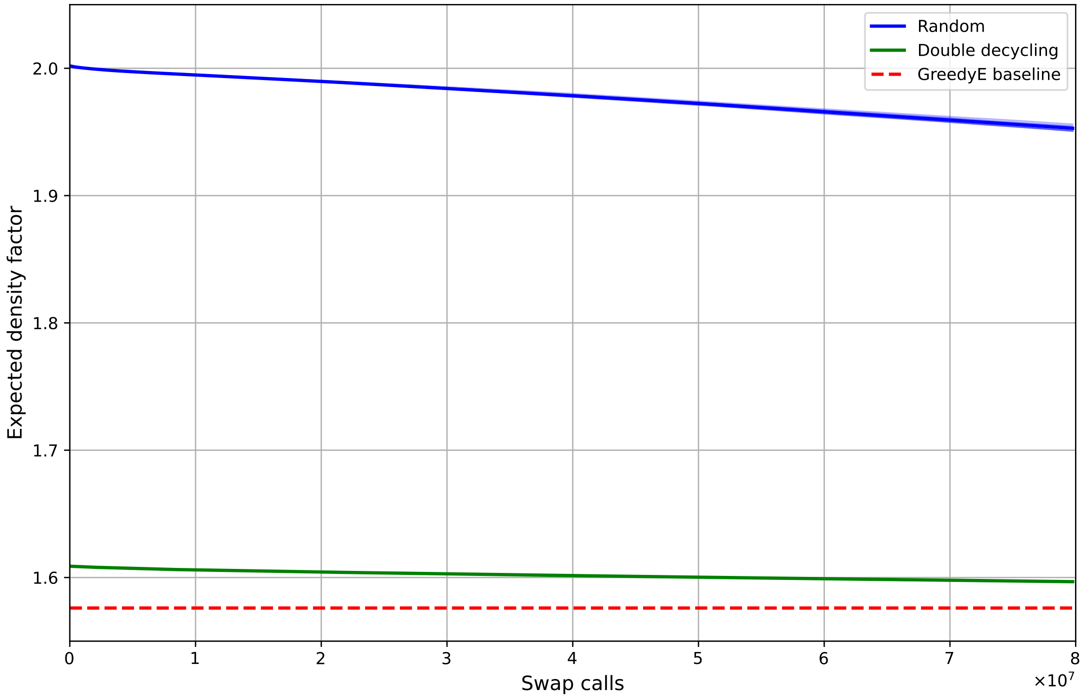

Fig. S7. Expected density factor for $k = w = 15$ and $\sigma = 2$ for a random order and a double-decycling order both swapped using SwapDFS. We compared their density factor to that of a baseline GreedyE order generated as part of GM-expected (which was selected among 4096 runs of GreedyE). We ran $8 \cdot 10^7$ steps of Swap (on our hardware, it took 15% more time than 4096 runs of GreedyE) on 8 independent cores. We report the average over the 8 cores as the change of density over time was very similar for all cores (with the maximum difference being less than 0.04%).
